# Therapeutic activity of modified U1 core spliceosomal particles

**DOI:** 10.1038/ncomms11168

**Published:** 2016-04-04

**Authors:** Malgorzata Ewa Rogalska, Mojca Tajnik, Danilo Licastro, Erica Bussani, Luca Camparini, Chiara Mattioli, Franco Pagani

**Affiliations:** 1 Human Molecular Genetics, International Centre for Genetic Engineering and Biotechnology, Padriciano 99, 34149 Trieste, Italy; 2 CBM S.c.r.l., Area Science Park, Basovizza, 34149 Trieste, Italy

## Abstract

Modified U1 snRNAs bound to intronic sequences downstream of the 5′ splice site correct exon skipping caused by different types of mutations. Here we evaluate the therapeutic activity and structural requirements of these exon-specific U1 snRNA (ExSpeU1) particles. In a severe spinal muscular atrophy, mouse model, ExSpeU1, introduced by germline transgenesis, increases SMN2 exon 7 inclusion, SMN protein production and extends life span. *In vitro*, RNA mutant analysis and silencing experiments show that while U1A protein is dispensable, the 70K and stem loop IV elements mediate most of the splicing rescue activity through improvement of exon and intron definition. Our findings indicate that precise engineering of the U1 core spliceosomal RNA particle has therapeutic potential in pathologies associated with exon-skipping mutations.

## Abstract (web summary)

Modification of the spliceosome is being tested as a potential therapy for exon-skipping diseases, such as spinal muscular atrophy (SMA). Here the authors show that 70K and stem loop IV structural elements of a modified U1 particle are essential for splicing enhancement and effective treatment of SMA mice.

## Graphical abstract

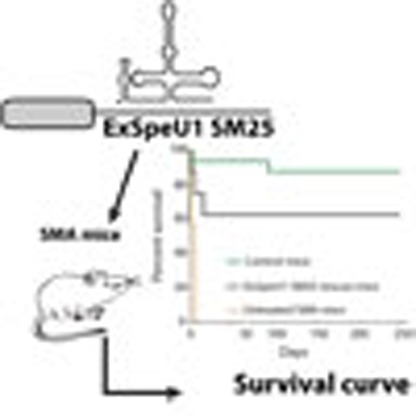

Five small ribonucleoprotein particles (U1, U2, U4, U5 and U6 snRNPs) and ∼150 proteins^1^ constitute the spliceosome, a macromolecular machinery involved in pre-messenger RNA (pre-mRNA) splicing. Within pre-mRNA, specific signals, such as the 5′ and 3′ splice sites, the branch point sequence and additional less conserved intronic or exonic elements with enhancer or silencer functions (intron splicing enhancer, exon splicing enhancer (ESE), intron splicing silencer (ISS), exon splicing silencer, respectively)^2^,^3^,^4^ drive the spliceosome to identify the correct exon–intron boundaries. The first step of spliceosome assembly involves the binding of the U1 snRNP to the 5′ splice sites of an exon through its 9-bp tail^5^. U1 snRNA is 165 bp long and has a stable and defined secondary structure that interacts with a set of U1-specific proteins, U1-A, U1-70K and U1-C, as well as with the Smith antigen (Sm) proteins, common to all the U-rich snRNAs^6^,^7^,^8^. The precise contribution of the U1 snRNP-associated proteins to normal splicing is not completely understood, but 70K is considered to have a major role. *In vitro*, the interaction between the RS-domain of 70K and RS-domain-containing splicing factors (SR proteins) bound in the exons^9^,^10^ promotes their selection for inclusion, a process known as exon definition^11^,^12^. U1C, in contrast to original predictions, is not essential for splicing as cells depleted of this factor have a preferential effect only on a narrow group of weakly defined exons^13^. The important U1 snRNA structural element stem loop IV interacts with the U2 snRNP-specific protein SF3A1, which is crucial for splicing as it promotes the alternative mechanism of splicing, namely ‘intron' definition^14^. Apart from an established role in splicing, U1 snRNP has an additional effect on transcription^15^ and on polyadenylation^16^,^17^,^18^. Both U1A and 70K are reported to affect polyadenylation^18^,^19^,^20^,^21^.

Exon skipping is one of the most frequent disease-causing splicing defects^22^. Different types of mutations at the pre-mRNA splice site consensus or in the accessory splicing regulatory elements can induce exon skipping. One of the most studied diseases involving exon skipping is spinal muscular atrophy (SMA), an autosomal recessive neuromuscular disease affecting α-motoneurons in the anterior horn of the spinal cord. SMA is caused by a homozygous loss-of-function of survival motor neuron (*SMN1*) gene, which encodes SMN, a key protein mainly involved in the biogenesis of snRNPs^6^. In the paralogous *SMN2* gene, a C>T transition disrupts and transforms an ESE^23^,^24^,^25^ into an exon splicing silencer^26^, inducing skipping of exon 7. As a result, in SMA-affected individuals, the reduced amount of normal transcript produced from *SMN2* only compensates partially for the loss of *SMN1*. An SMA mouse model, which integrates different copies of the human-specific *SMN2* gene, mostly reproduces the disease phenotype^27^. This is one of the very few available animal models in which the same exon skipping defect is associated with a disease in humans and mice.

In the past decade, different strategies have been developed to rescue exon skipping defects and for the most part the SMA mouse model has been used. These approaches include antisense oligonucleotides or U7-derivatives^28^,^29^,^30^,^31^ that interfere with splicing-regulatory elements^31^,^32^,^33^,^34^,^35^. Other approaches include the recruitment of positive splicing factors^36^ and the use of chemical compounds^37^,^38^. We developed an approach to correct exon skipping, in different diseases, based on the modification of the core spliceosomal U1 snRNA^39^,^40^,^41^. The modified small RNAs, named exon-specific U1s (ExSpeU1s), have engineered 5′-tails that direct their loading onto intronic regions downstream of 5′ (donor-) splice sites and rescue exon skipping in coagulation factor IX exon 9, CFTR exon 13, SMN2 exon 7 and SPINK5 exon 7 (ref. 39). The positive effect on pre-mRNA splicing improves the protein function in patient-derived primary cells^40^,^41^ and *in vivo*, ExSpeU1 expression mediated by AAV delivery efficiently rescues aberrant splicing^40^. Interestingly, a unique ExSpeU1 is active on different types of mutations. Apart from canonical GU and AG dinucleotides at the splice sites, ExSpeU1 correct mutations in the 5′ splice sites consensus, in exonic regulatory elements or in polypyrimidine tract of the 3′ splice site, suggesting that some component(s) of the particle is/are involved in the splicing rescue activity^39^.

Here, we investigate the therapeutic splicing activity and structural requirements of the modified core spliceosomal U1 snRNPs. Germline expression of the active particle does not result in obvious phenotypic abnormalities and crossing of an ExSpeU1 transgenic animal with a severely affected SMA model mouse considerably improves the phenotype and survival. Using ExSpeU1 mutants and silencing experiments in splicing assays on different gene systems, we show that the splicing rescue activity of these U1-like particles is dependent on the U1 snRNP 70K protein and on the loop IV structure of the U1snRNA, but not on the U1A protein. Altogether, the experimental evidence indicates that precise engineering of one of the core components of the spliceosome efficiently corrects defective exons and has a significant therapeutic activity in a splicing-defective mouse model.

## Results

### SMN splicing and protein rescue in SM25 transgenic SMA mice

**Figure 1:**
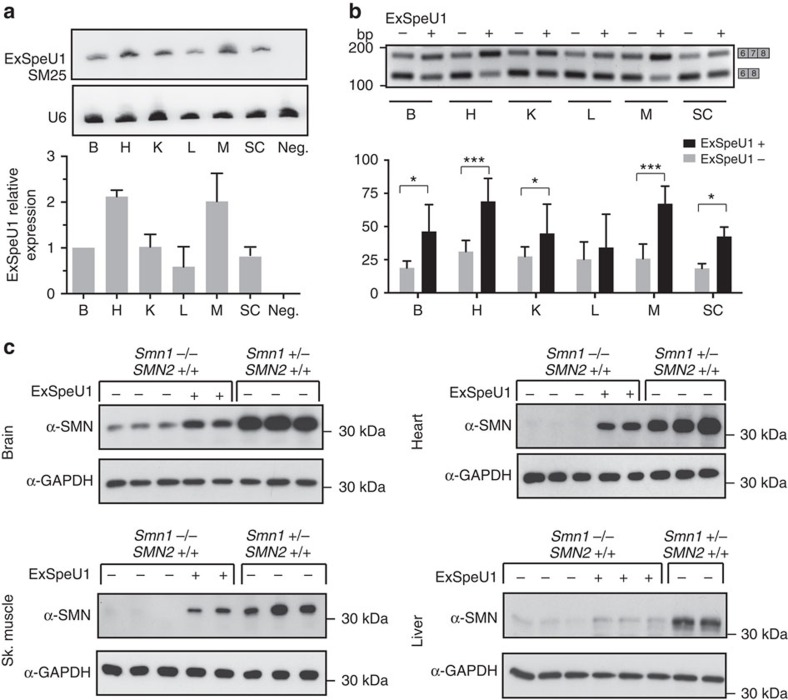
SMN splicing and protein rescue in SM25 transgenic SMA mice. (**a**) Northern blot analysis of ExSpeU1 SM25 expression in *Smn1* heterozygous mice at P40 (B, brain; H, heart; K, kidney; L, liver; M, muscle; SC, spinal cord). ExSpeU1 expression levels are normalized to U6 snRNA and brain is set to 1. Data are the mean±s.e.m. of four animals except for SC (three mice). ‘Neg.' denotes ExSpeU1−/− mice. (**b**) *SMN2* specific splicing assay in *Smn1* heterozygous mice at P40. Data represent the mean±s.e.m. in eight ExSpeU1 +/− and nine ExSpeU1 −/− animals except for SC (three mice; Student's *t*-test *P≤0.05, ***P≤0.001). Bars on the right represent SMN2 exon 7 inclusion and exclusion splicing isoforms. (**c**) SMN protein expression levels detected by western blotting in SMA-affected (Smn1−/−, SMN2+/+), SMA-rescued (Smn1−/−, SMN2 +/+; ExSpeU1 +/−), and control animals (Smn1+/−, SMN2+/+, ExSpeU1+/−) at P2–3.

Binding of ExSpeU1s in intronic sequences downstream of the 5′ splice sites rescues exon skipping caused by different types of mutations^39^,^40^,^41^. To evaluate their therapeutic potential, we focused on SMA and created a transgenic mouse line with germline-integrated SM25, an ExSpeU1 that efficiently promotes SMN2 exon 7 inclusion^39^,^40^. By northern blot analysis, all the tissues tested expressed SM25, with higher levels detected in the heart and muscle, and lower in the liver (Fig. 1a). Mapping of the transgene^42^ identified one integrated copy of ExSpeU1 cassette in chromosome 1. Since the SM25 construct was prepared to specifically improve *SMN2* exon 7 definition, we crossed the SM25 transgenic line with a severe SMA mouse model, FVB.Cg-Tg(SMN2)89Ahmb *Smn1*^*tm1Msd*^/J that show symptoms and neuropathology similar to SMA type I patients and survive 4–6 days. We performed PCR with reverse transcription (RT–PCR) on RNA samples from mice at postnatal day 40 (P40) to examine *SMN2* splicing changes in various tissues caused by ExSpeU1 expression. In most of the tissues analysed, *Smn1* heterozygous mice carrying human *SMN2* gene along with the ExSpeU1 transgene (Smn1+/−, SMN2+/+,ExSpeU1+/−) showed a significant increase in the percentage of *SMN2* exon 7 inclusion. Consistent with the SM25 expression data, the highest SMN2 exon 7 inclusion levels were observed in the heart and muscle with ∼70% inclusion. The brain, spinal cord and kidney had intermediate levels (∼40–50%), and liver had a small improvement (Fig. 1b). Moreover, we have tested SMN protein expression from SMA-affected (Smn1−/−, SMN2+/+), SMA-rescued (Smn1−/−, SMN2+/+; ExSpeU1+/−) and control animals (Smn1+/−, SMN2+/+, ExSpeU1+/−) by immunoblotting. Since SMA-affected mice without ExSpeU1 survive only up to 6 days, we performed protein analysis at P2–3 (Fig. 1c). In comparison with control animals, the affected SMA mice showed a significant reduction of SMN expression (Fig. 1c and Supplementary Fig. 1e) One copy of the ExSpeU1 restored SMN protein expression to ∼30% in the brain, spinal cord, skeletal muscle and the heart, and to ∼10% in liver (Fig. 1c and Supplementary Fig. 1). These data indicate that stable integration of one copy of SM25 ExSpeU1 increases SMN2 exon 7 inclusion and SMN expression in most of the tissues analysed.

### SM25 expression prolongs the lifespan of SMA-affected mice

**Figure 2:**
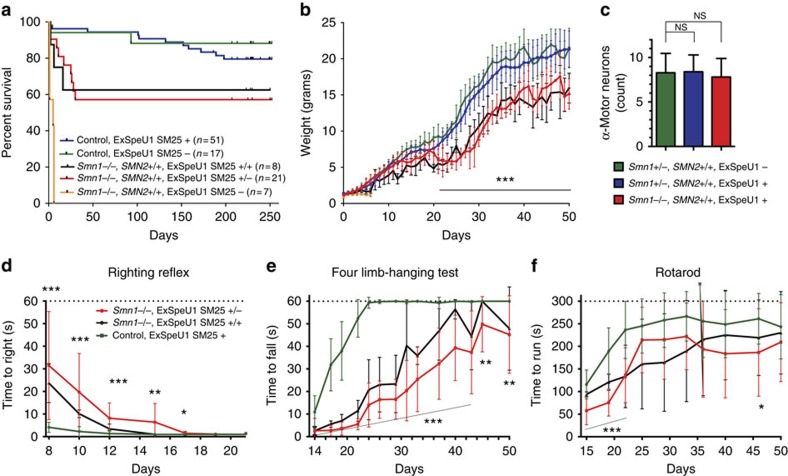
Improved phenotype and survival in SM25 transgenic SMA mice. (**a**) Kaplan–Meier survival curves from P0 to P250. Control ExSpeU1− corresponds to Smn1+/+, SMN2+/+ ExSpeU1−/− genotype; control ExSpeU1+ include hemizygote (Smn1+/+, SMN2+/+, ExSpeU1+/−, *n*=40) and homozygote (Smn1+/+, SMN2+/+, ExSpeU1+/+, *n*=11) animals; Smn1−/−, ExSpeU1− are SMA-affected mice. SMA-rescued are divided into hemizygote (Smn1−/−, SMN2+/+; ExSpeU1+/−) and homozygote (Smn1−/−, SMN2+/+; ExSpeU1+/+) mice. (**b**) Body weight from P1 to P50. (**c**) Number of α-motor neurons in SMA-rescued animals and controls at P40. (**d**) Righting reflex between P8 and P21; Control (*n*=18), Smn−/−, SMN2+/+, ExSpeU1+/− (*n*=18), Smn−/−, SMN2+/+, ExSpeU1+/+ (*n*=3). (**e**) Four limb-hanging test in mice between P14 and P50: SMA-rescued mice Smn−/−, SMN2+/+, ExSpeU1+/− (*n*=17) and Smn−/−, SMN2+/+, ExSpeU1+/+ (*n*=3) show less ability than control animals (*n*=17) to hang on the grid. (**f**) Rotarod test in mice from P15 to P50. Rescued-SMA mice Smn−/−, SMN2+/+, ExSpeU1+/− (*n*=14), Smn−/−, SMN2+/+, ExSpeU1+/+ (*n*=3) stayed less time on the rotarod than the control group (*n*=14); although after P22, there was no significant difference between the groups. Values between control and Smn−/−, SMN2+/+, ExSpeU1+/− mice are shown as mean±s.d. Student's *t*-test *P≤0.05, **P≤0.01, ***P≤0.001; NS, not significant.

To evaluate the phenotypic improvement of the increased SMN protein expression, we analysed survival of SMA-affected mice, SMA-rescued animals and their healthy siblings. Strikingly, expression of the SM25 U1-like particle extensively prolongs the survival of SMA mice: whereas the affected animals, representing a very severe form of the disease, survive only 4–6 days, ∼60% of rescued mice are alive at P250, the last day of recording (Fig. 2a). Most of the mortality was observed during the first 5 weeks of life and no significant differences were observed between hemizygote and homozygote SM25 transgenic animals (Fig. 2a). The increase in survival in the rescued mice was associated with a significant improvement in weight, although the animals gain weight slower than normal mice (Fig. 2b). At P50, SMA-rescued mice had ∼70% of the heterozygous littermates' weight. Nevertheless, they were able to breed and deliver the litter, although we observed an increased mortality of the pups delivered by the SMA-rescued females, which was probably due to reduced capacity to feed the pups. Since the motor neuron degeneration is the most important hallmark of SMA pathology, we also evaluated the α-motor-neurons count in SMA-affected and control mice. The α-motor-neuron counts in the spinal cords of SMA-rescued mice were comparable to the control heterozygous littermates with no significant difference between these groups (Fig. 2c). In functional analysis, rescued-SMA mice had prematurely reduced ability to perform righting reflex, four-limb hanging test and Rotarod compared with control animals (Fig. 2d–f). However, consistent with a delayed development of the neuromuscular performance, the rescued mice progressively recover the defects during development and performed the tests equally well as control mice when older. In comparison with hemizygote mice, homozygote SM25 transgenic animals performed slightly better for righting reflex and four-limb hanging test but this difference did not reach the statistical significance (Fig. 2d,e). The rescued mice had no obvious phenotypic abnormality at P80, apart from reduced weight.

### Selectivity of the splicing changes induced by SM25

**Figure 3:**
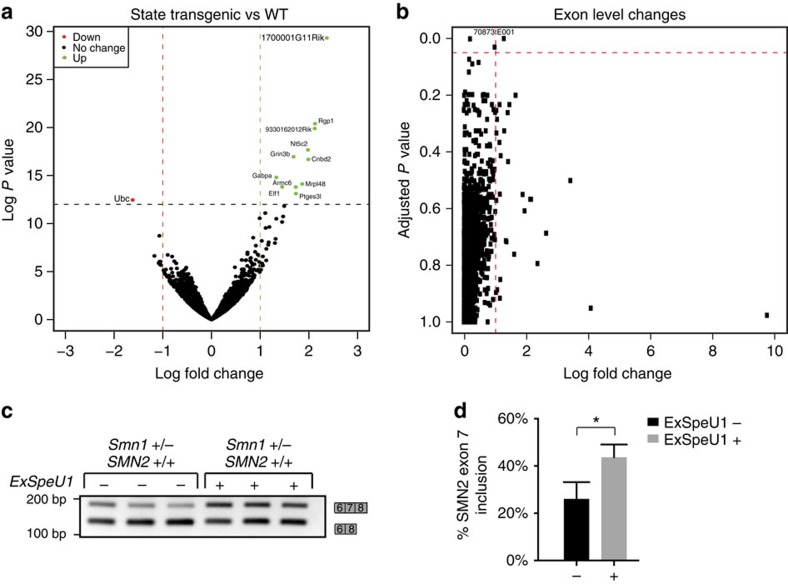
ExSpeU1 SM25 has a minimal genome-wide effect in spinal cord. (**a**) Difference in total transcript expression in the spinal cord. Volcano plots for changes in gene expression between wild-type (Smn1+/−, SMN2 +/+; ExSpeU1−/−) and SM25 ExSpeU1 transgenic animals (Smn1+/−, SMN2+/+, ExSpeU1+/−; *n*=6 with three samples per group). Horizontal and vertical dashed lines indicate cutoff values (FDR value of 0.05 and absolute logarithmic fold change >2). Genes having a significantly altered expression are emphasized in red and green. (**b**) Difference in expression of individual exons in the spinal cord in Mus Musculus transcripts. The plot shows exon-level changes of all exons obtained from UCSC mm10 RefGene. The horizontal axis shows variation (log_2_-fold) in expression between wild-type (WT) and SM25 ExSpeU1 transgenic animals while the vertical axis refers to *P* value after Benjamini–Hochberg correction. (**c**) *SMN2*−specific splicing assay in the spinal cord of WT and transgenic animals. Bars on the right represent SMN2 exon 7 inclusion and exclusion splicing isoforms. (**d**) Data obtained from **c** represent the mean±s.e.m. of SMN2 splicing changes in three WT and three transgenic animals (Student's *t*-test *P≤0.05).

To explore the selectivity of ExSpeU1, we measured global gene expression changes in the spinal cord of wild-type (Smn1+/+, SMN2+/+, ExSpeU1−/−) and SM25 ExSpeU1 transgenic (Smn1+/+, SMN2+/+, ExSpeU1+/−) animals by mRNA-Seq. We only observed altered expression of a small proportion of the 12,414 investigated genes, indicating that SM25 ExSpeU1 does not have a widespread effect on gene expression (Fig. 3a and Supplementary Fig. 2a). We identified one gene that was downregulated and 11 genes that were upregulated. We validated 6 out of 12 transcript changes using quantitative RT–PCR experiments (Supplementary Fig. 2b); in all the remaining cases that did not reach the statistical significance, the changes were consistent with the RNA-Seq data. To evaluate the effect on specific pathways, we tested all the differentially expressed genes by Ingenuity Pathway Analysis (Qiagen). Canonical pathway and network analysis showed no significant enrichment (score <4). To assess the possible large-scale effect of SM25 on splicing, we measured the U1/U6 snRNA ratio and analysed selected constitutive and alternative splicing events. SM25 had no effect on the endogenous U1/U6 snRNA ratio (Supplementary Fig. 3) indicating that ExSpeU1s do not affect the abundance of core spliceosomal snRNAs. We then evaluated selected alternative splicing events previously reported to change in the spinal cord of SMA mice and potentially related to the disease pathology^43^. Apart from the expected rescue of transgenic human SMN2 exon 7 (Fig. 3b), we observed no changes in nine constitutive and nine alternative splicing events in the spinal cord by RT–PCR (Supplementary Fig. 4). Similarly, the evaluation of RNAseq data for splicing alterations at the exon level, revealed only one candidate event (*Cnbd2*) where splicing differs in the SM25-expressing tissue (Fig. 3b). The gene identified in the splicing alterations, *Cnbd2*, is also included in the list of 12 genes whose expression changes. Collectively these results suggest that SM25 ExSpeU1 expression does not induce widespread changes in gene expression or splicing.

### ExSpeU1s are assembled as U1-like particles

**Figure 4:**
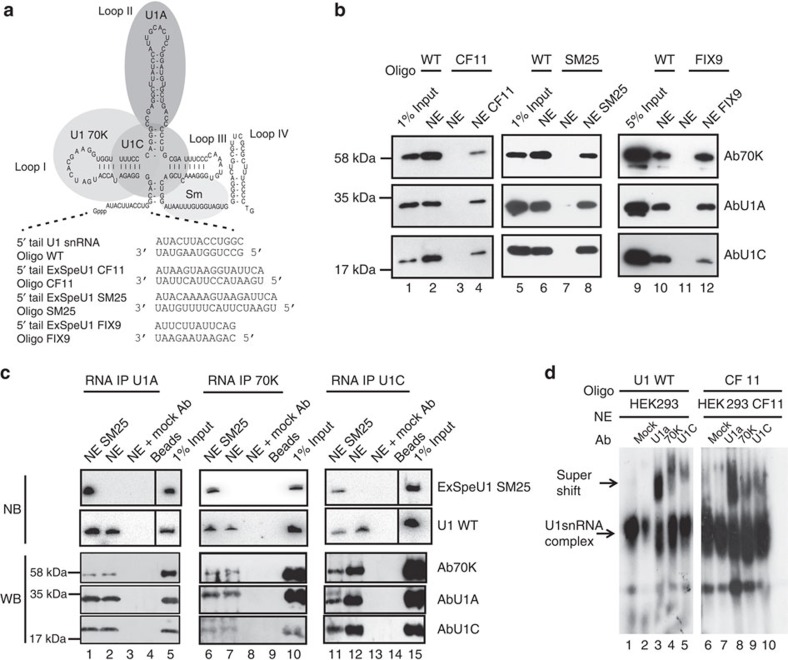
Protein composition of ExSpeU1s and endogenous U1 snRNP. (**a**) Schematic representation of U1 snRNA secondary structure and associated proteins along with the RNA oligonucleotides used in affinity purification, RIP and EMSA. (**b**) Affinity purified CF11, SM25 and FIX9 ExSpeU1s contain 70K, U1A and U1C. Nuclear extracts (NE) from Hek293 cells, transfected with the indicated ExSpeU1s, or not transfected cells were incubated with the corresponding biotinylated 2′-*O*-methyl-RNA oligonucleotides. Affinity-purified snRNPs were analysed by western blotting using antibodies against U1–70K, U1A and U1C. (**c**) RNA-immunoprecipitation analysis of SM25 ExSpeU1. Hek293 NEs from cells transfected with ExSpeU1 SM25 or not transfected cells were incubated with antibodies against U1A, 70K and U1C. RNAs and proteins purified from the RIP complexes were analysed by northern and western blotting, respectively with the indicated probes/antibodies. Mock Ab corresponds to anti-tubulin. (**d**) CF11 ExSpeU1 snRNP has the same electrophoretic mobility as normal U1 in EMSA. Radiolabelled RNA oligonucleotides complementary to normal U1 (lanes 1–5) or ExSpeU1 CF11 (lanes 6–10) were incubated with nuclear extracts transfected with ExSpe CF11 or mock (not transfected cells). Addition of the indicated antibodies super-shifted the complexes in U1wt and ExSpe CF11. Control EMSA experiments are shown in Supplementary Fig. 5.

To understand how ExSpeU1 promotes definition of defective exons, we evaluated the structural requirement of SM25 along with CF11 and FIX9, two previously reported ExSpeU1s, which are active on CFTR exon 13 and FIX exon 5, respectively^39^,^40^. We investigated the composition of the resulting particles on these ExSpeU1s, using RNA oligonucleotides that distinguish ExSpeU1s from endogenous U1 snRNP (Fig. 4a). We performed affinity purification, RNA immunoprecipitation (RIP) and EMSA experiments using nuclear extracts obtained from the cells transfected with different ExSpeU1s. In the affinity purification assays, we pulled down ExSpeU1 snRNPs using a biotinylated 2′-*O*-methyl-RNA oligonucleotide complementary to their 5′ tail. Associated proteins were identified by western blotting using antibodies against U1A, 70K and U1C. Experiments showed that the ExSpeU1 snRNPs contain the U1-specific proteins U1A, 70K and U1C (Fig. 4b). As expected, in untransfected cells, the ExSpeU1-specific oligonucleotides did not precipitate endogenous U1 snRNPs (Fig. 4b, lanes 3, 7 and 11). To further characterize the snRNP composition, we performed an RIP experiment with SM25 (Fig. 4c). HEK 293 nuclear extracts from the cells transfected with ExSpeU1 SM25 were incubated with agarose beads coated with U1A, 70K and U1C Ab and bound RNA was identified by northern blotting. The membranes were hybridized with oligonucleotides complementary to the 5′ tail of ExSpeU1 SM25 or with U1 snRNA. As expected, in mock-transfected nuclear extracts, immunoprecipitated RNAs only hybridizes with U1 snRNA (Fig. 4c, lanes 2, 7 and 12). In cells transfected with ExSpeU1 SM25 (lanes 1, 6 and 11), U1A, 70K and U1C antibodies immunoprecipitated both ExSpeU1 SM25 and U1 snRNAs indicating that these three proteins are present in the ExSpeU1 particles. Control analysis of associated proteins by western blotting showed the correct presence of the U1 specific proteins in the IP (Fig. 4c, lower panels). Last, we evaluated the ExSpeU1 snRNP composition in EMSA. In this assay, only the CF11 resulted in the formation of a specific complex (Supplementary Fig. 5). It is possible that the lack of complex formation in the other cases might be due to the sequence composition of the 5′ tails, which, in the experimental condition of the assay, is not accessible or does not have the optimal hybridization condition for the oligonucleotides. The analysis of CF11 ribonucleoparticles showed that it has the same mobility as the U1 snRNP and is super-shifted by U1A, U1-70K and U1C antibodies (Fig. 4d). Thus, using three different assays, affinity purification, RIP and EMSA, we demonstrate that ExSpeU1 snRNPs form particles that resemble U1 snRNP and contain the U1-specific proteins U1A, 70K and U1C.

### ExSpeU1s do not require endogenous U1 snRNP

To understand whether these U1-like particles affect the splicing rescue in a U1 snRNP-dependent manner, we decreased the amount of functional endogenous U1 snRNP. To this aim, we used a U1 decoy (D1) that covers the 5′ end of normal U1 RNA and blocks its binding to the 5′ splice sites^44^. In SMN2, using this approach, we showed that ExSpeU1-mediated splicing rescue, in contrast to the antisense masking of the ISS, does not require U1 snRNP, suggesting that these two molecules improve exon definition by different mechanisms^40^. To establish the amount of U1 snRNP sequestered by the decoy in transfection experiments, we performed an RNase H protection assay. Extracts from the cells transfected with a control D3 plasmid or with the decoy D1 plasmid were incubated with RNase H and an antisense DNA oligonucleotide that is complementary to the 5′ end of U1 snRNA. In this experimental condition, D1 transfection, but not control D3, functionally protected ∼70% of endogenous U1 snRNP (Supplementary Fig. 6a), which presumably correspond to the sequestered fraction. We co-transfected splicing minigenes along with U1 decoy and ExSpeU1s to evaluate if the ExSpeU1s-mediated splicing rescue requires U1 snRNP. We analysed the effect of the decoy on wild-type and mutated SMN, FIX and CFTR minigenes. In all the cases, the U1 decoy (D1) did not affect the splicing rescue induced by ExSpeU1s (Supplementary Fig. 6b). This demonstrates that ExSpeU1s binding to the intronic sequences downstream of the 5′ splice sites do not require additional U1 snRNP for splicing enhancement.

### ExSpeU1 mutants with altered binding properties

**Figure 5:**
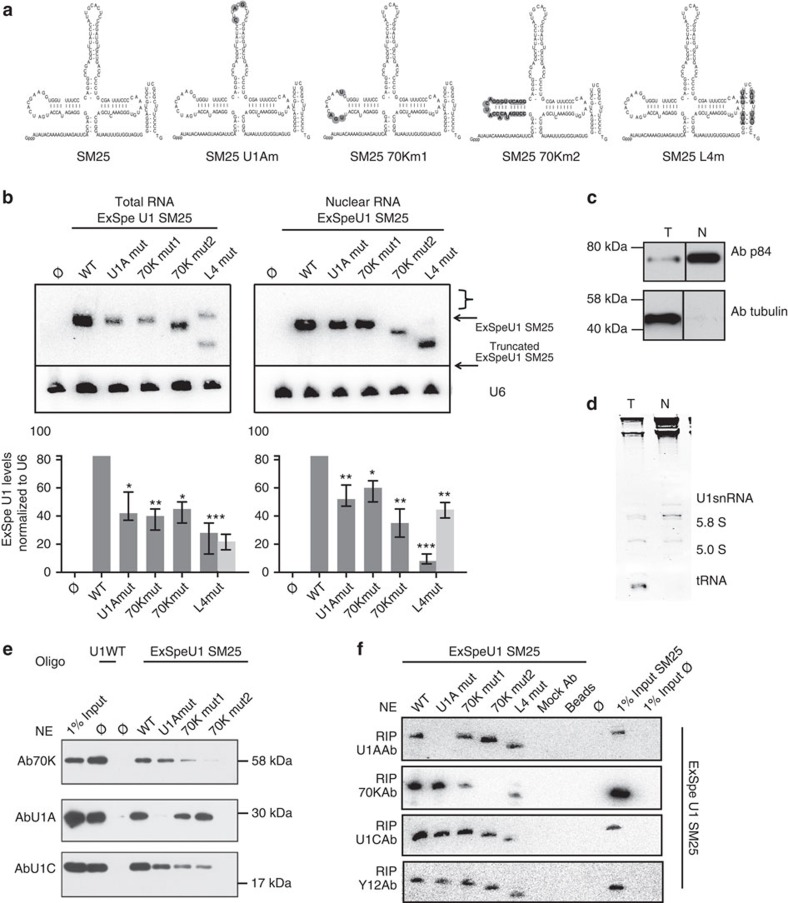
ExSpeU1s SM25 mutants with defective protein binding. (**a**) Schematic representation of RNA secondary structures and sequences of SM25 ExSpeU1 mutants. Highlighted modified nucleotides are shown in grey. (**b**) Expression levels, quality and nuclear distribution of the ExSpeU1 mutant RNAs. Representative northern blot analysis of ExSpeU1 mutants in total and nuclear RNA fractions. RNA was probed for ExSpeU1 SM25 and for U6 and the histograms below each northern blot show the ExSpeU1 SM25 variants' expression levels relative to U6. Values are the mean±s.e.m. of four independent experiments; *P≤0.05, **P≤0.01, ***P≤0.001. (**c**,**d**) Purity of total (T) and nuclear (N) fractions assessed by WB using Human Nuclear Matrix Protein p84, tubulin Ab and by EtBr staining of sRNAs loaded on 8% urea– polyacrylamide gel electrophoresis gel. Transfer RNA (tRNA) is missing in the nuclear fraction. (**e**) Affinity purification of ExSpeU1 SM25 mutants. Nuclear extracts (NE) from Hek293 cells transfected with the indicated constructs were incubated with the SM25 biotinylated oligonucleotide. Affinity-purified snRNPs were analysed by WB using antibodies against U1–70K, U1A and U1C. (**f**) RNA-IP analysis of SM25 ExSpeU1 variants. Hek293 NE from cells transfected with ExSpeU1 SM25 variants or not trasfected cells were incubated with antibodies against U1A, 70K, U1C and Sm proteins. RNAs purified from the RIP complexes were analysed by northern blotting. Pulled-down complexes were also analysed by northern blotting with U1 wild-type probe (Supplementary Fig. 9).

To identify the structural component of ExSpeU1 involved in splicing rescue, we evaluated the particles expressed from SM25 RNA mutants. We evaluated two previously described mutations in loop I and II (U1Amut and 70Km1), which *in vitro* do not bind to 70K and U1A^19^,^45^, respectively and a more disruptive variant, 70Km2, in which we modified the entire stem-loop II structure (Fig. 5a). The U1Am and 70Km1 mutants were previously tested in complementation assays, but their effect on the resulting particle was never analysed *in vivo*^15^,^19^,^46^,^47^. We also considered a variant in stem loop 4 (SL4m)^14^ (Fig. 5a), as this structural element has been recently reported to be essential for splicing *in vivo* because of its interaction with the U2 snRNP-specific SF3A1 protein. The cells were transfected with the SM25 ExSpeU1 variants and total and nuclear U1 RNAs analysed by northern blotting. In comparison to the native ExSpeU1, all mutations reduced (to ∼40–50%) the amount of total and nuclear RNAs, indicating that *in vivo* they lack some structural components that reduce, but do not completely disrupt the biosynthesis, assembly and nuclear import of the particles (Fig. 5b). In addition, the SL4 mutant produced a truncated ExSpeU1 RNA that was preferentially located in the nuclear fraction (Fig. 5b). Consistent with the fact that defective SL4 variants are processed to generate a truncated shorter isoform, as reported^48^, we show by 3′-RNA adaptor ligation that in our case the shorter U1 variant is missing the last 22–24 nucleotides (Supplementary Fig. 7) and accordingly maintains the consensus Sm binding site. We next evaluated the composition of the ExSpeU1 snRNP by affinity purification. Native and mutant ExSpeU1 snRNPs were affinity purified and protein composition was evaluated by western blotting. In comparison with the corresponding native counterparts, U1Am and the 70Km2 variants specifically lack U1A and 70K binding, respectively (Fig. 5e). In contrast, the less-disruptive loop I substitutions in 70Km1, previously used in complementation assays^15^, did not completely abolish the interaction with 70K (Fig. 5e). We confirmed the binding properties of the three mutants on a different ExSpeU1, CF11 (Supplementary Fig. 8). To further validate these results, we performed RNA-IP experiments (Fig. 5f). In this case, U1A Ab immunoprecipitated all ExSpeU1 RNAs except the U1Am1 mutant; 70K Ab failed to pull down the 70Km2 variant whereas U1C and Y12 immunoprecipitated all ExSpeU1 RNAs (Fig. 5f). RIP efficiency was confirmed by re-probing the membranes for endogenous U1 (Supplementary Fig. 9). Thus, *in vivo* the two U1Am and 70Km2 mutants specifically inhibit U1A and 70K binding, respectively. The SL4 mutant showed a slight reduction in the amount of RNA pulled down by the 70K and U1C Ab and no effect on RNA pulled down by Y12 (Fig. 5f). This suggests that the SL4 structure might contribute to 70K and U1C binding. Interestingly, U1C is present on all variants suggesting that neither, 70K, U1A nor the stem loop 4, are strictly required for its recruitment on the mature particle. However, consistent with previous studies that indicate an important role for 70K in mediating association of U1C to the particle^7^, we observed a reduced amount of this protein in the 70Km2 mutant.

### Proteins involved in ExSpeU1-mediated splicing rescue

**Figure 6:**
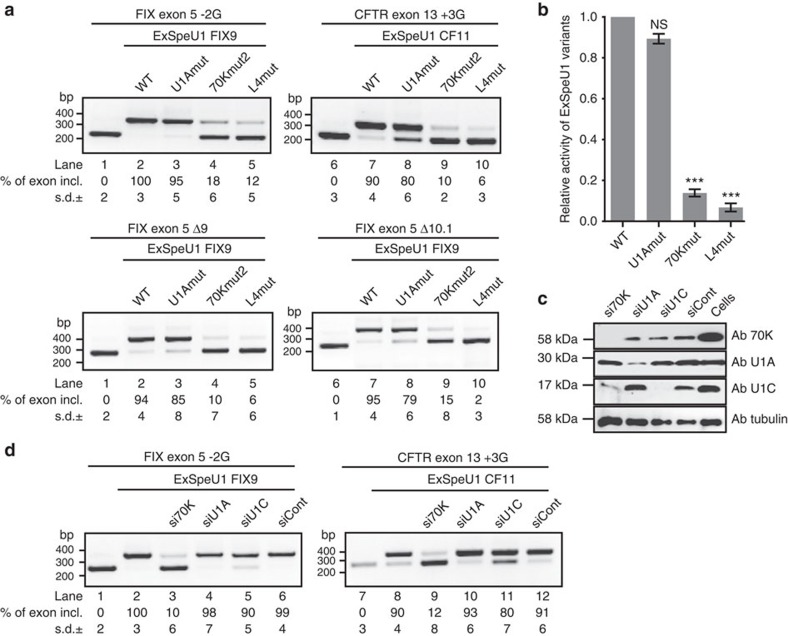
Role of 70K protein and stem loop 4 in severely affected exons. (**a**) Effect of ExSpeU1 variants on mutant minigenes. Minigenes were co-transfected with the ExSpeU1 variants and the splicing pattern was evaluated by RT–PCR. Percentage of exon inclusion was quantified by ImageJ. Data represent the means±s.d. of three independent experiments performed in duplicate. (**b**) The histogram shows the mean activity of the ExSpeU1 mutants relative to the corresponding native particles calculated as mean of the four minigene systems. For each ExSpeU1 variant, the difference between the basal and induced exon inclusion was calculated as Δψ. Values obtained for WT particles were set to 1 and then used to calculate the relative activity of each ExSpeU1 mutant. Data represent the mean±s.e.m. of ExSpeU1 mutants relative activity from four minigene systems (Student's *t*-test ***P≤0.001; NS, not significant). (**c**) Effect of silencing of U1-specific proteins on native ExSpeU1-induced splicing rescue. The efficacy of siRNA treatments on the endogenous protein levels was verified by WB using antibodies against U1A, 70K, U1C. Tubulin was used for normalization. (**d**) siRNA-treated and control HeLa cells were co-transfected with minigenes and corresponding ExSpeU1s as indicated. The splicing pattern was evaluated by RT–PCR. Data are shown as means±s.d. of three independent experiments performed in duplicate.

**Figure 7:**
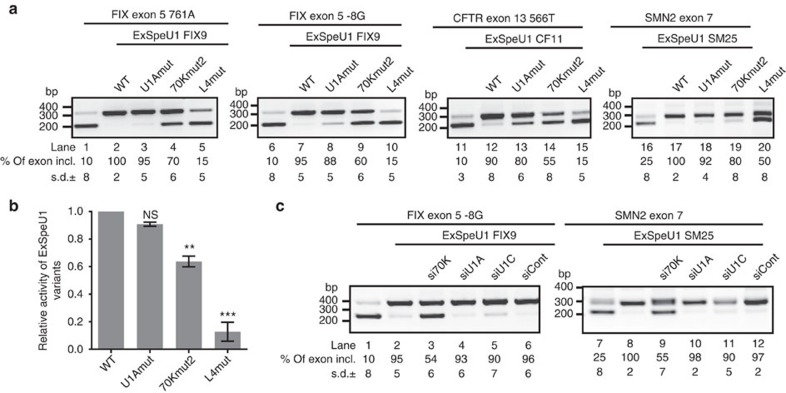
The U1 70K protein is partially dispensable in mildly affected exons. (**a**) Effect of ExSpeU1 variants on mutant minigenes. Splicing patterns were evaluated and quantified as in Fig. 6. Data are expressed as means±s.d. of three independent experiments performed in duplicate. (**b**) The histogram shows the activity of the ExSpe U1 mutants relative to the native particles and data are calculated as described in Fig. 6. Data represent the mean of the effect in four minigene systems. (Student's *t*-test **P≤0.01, ***P≤0.001; NS, not significant). (**c**) Effect of silencing of U1-specific proteins on native ExSpeU1-induced splicing rescue. siRNA treated and untreated HeLa cells were co-transfected with minigenes and corresponding ExSpeU1s. Data are expressed as mean±s.d. of three independent experiments done in duplicate.

To understand the contribution of the U1-specific proteins to splicing, we performed a complementation assay using native ExSpeU1 and loop I, II and SL4 variants. We initially considered the effect of ExSpeU1s on mutations that severely affect exon definition. Of the previously reported ExSpeU1 rescued variants, we selected four disease-causing mutations that severely affect exon inclusion: +3G and −2G at the 5′ splice sites consensus in CFTR exon 13 and FIX exon 5, respectively and two exonic deletions in FIX exon 5 (Δ9 and Δ10.1). To ensure comparable expression levels of the different ExSpeU1s in our complementation experiments, we co-transfected an increased amount of the mutants relative to the native ExSpeU1s and equivalent levels of RNAs were verified by northern blotting (Supplementary Fig. 10). In all minigenes, U1Am had a rescue activity comparable to corresponding native ExSpeU1s, indicating that the presence of the U1A protein in the particle is dispensable for splicing (Fig. 6). In contrast, the SL4 mutant did not significantly rescue splicing (Fig. 6a,b, *P*<0.001, Student's *t*-test). Interestingly, the 70Km2 mutant failed to rescue exon skipping (Fig. 6a,b *P*<0.001, Student's *t*-test), indicating that the 70K protein in the ExSpeU1 particle is crucial for exon definition. To better investigate the role of the U1-specific proteins on the ExSpeU1-mediated splicing rescue, we performed silencing experiments of U1A, 70K and U1C. siRNA treatment resulted in nearly complete depletion of 70K and U1C and ∼80% reduction of U1A (Fig. 6c). As expected, 70K silencing caused a reduction in U1C^13^. Depletion of U1A and U1C did not significantly affect the ExSpeU1-mediated splicing enhancement (Fig. 6d, lanes 4, 5, 10, 11). In contrast, 70K silencing almost completely abolished the splicing enhancement induced by ExSpeU1s (Fig. 6d, lanes 3 and 9). We next evaluated less-disruptive mutations, to better understand the relationship between ExSpeU1 snRNP and exon definition. We considered mutations in exonic elements or in the polypyrimidine tract that maintain low levels of normal splicing (from 10 to 25%) (Fig. 7)^39^. For the FIX, CFTR and SMN2 minigenes, we evaluated the effect of ExSpeU1 variants along with silencing of the three U1-specific splicing factors. Native ExSpeU1s or U1Am rescued exon skipping (Fig. 7a,b) with equal efficiency, whereas SL4 did not considerably impact splicing (Fig. 7a,b *P*<0.001, Student's *t*-test). SL4 showed only a minor effect on the SMN2 exonic mutant (Fig. 7a lane 20), which was the least disruptive among the mutations tested. This small effect could either be due to a residual splicing-rescue activity of the defective particle in this particular context or to a partial antisense interference of the inactive SL4 mutant at the previously described nearby ISS-element^49^. The particle lacking 70K showed some reduced activity on partially defective exons (Fig. 7a). In comparison with the corresponding native ExSpeU1s, 70Km2 had ∼60% less activity (Fig. 7b, *P*<0.01, Student's *t*-test). Similarly, silencing of the U1 specific factors showed that U1A and U1C depletion had no significant effect on the ExSpeU1-mediated splicing enhancement. However 70K silencing reduced the ExSpeU1-mediated rescue to ∼55% (Fig. 7c). Thus, in the case of mutations that cause severe exon skipping, the ExSpeU1 snRNP-associated 70K protein (but not U1A and U1C) is essential for splicing rescue. However, the ExSpeU1 particle, devoid of 70K, retains some activity in those mutations that do not completely disrupt exon definition. Mutations in SL4, previously reported to affect binding of the U2 snRNP-specific SF3A1 protein^14^, produced an inactive ExSpeU1.

## Discussion

Several strategies have been developed to correct inherited splicing defects. Most of them aim at interfering with splicing regulatory elements using antisense oligonucleotides^31^,^32^,^33^,^34^,^35^, or have used antisense oligonucleotides to recruit positive splicing factors on defective exons^36^ but none has tested *in vivo* the possibility to directly modifying or regulating the core spliceosomal components. U1 snRNP is a core macromolecular spliceosomal component that is built around an RNA backbone, U1 snRNA. We show here that modifying U1 snRNA to bind specific intronic sequences downstream of the 5′ splice sites results in the assembly of a target-specific therapeutic U1-like particle (ExSpeU1). This modified U1 snRNP restores spliceosomal activity on several skipped exons, mainly through its associated 70K protein and stem loop IV. This effect does not require endogenous U1 snRNP. Most importantly, the splicing correction results in significant rescue of the phenotype in a very severe SMA mouse model, providing the first proof of principle *in vivo* of safety and efficacy of this novel therapeutic strategy.

To our knowledge, this is the first time that a specific modification of a core spliceosomal component has been shown to be safe and active in a mouse model. Our results show that germline expression of the U1-like SM25 particle is highly specific for processing of *SMN2* pre-mRNA and has no apparent side effects. This indicates that the expression of a U1-like particle that contains the SM25 sequence complementary to part of the SMN2 intron is not toxic *in vivo*. Strikingly, ExSpeU1 SM25 expression has a profound effect on the phenotype. Although the severe SMA mice die before P6 and show some embryonic lethality^50^, ∼60% of transgenic SM25 animals are alive after 250 days of observation (Fig. 2a). The significant rescue of the phenotype, splicing and protein observed with a single integrated copy of ExSpeU1 highlights the potential therapeutic activity of modified U1 snRNAs that bind in introns. The high rescue efficiency we observed, here *in vivo* and previously in cell culture^40^, can be due to the combined effect on exon and intron definition mediated by the structural 70K and SL4 elements, respectively. Even if the phenotypic improvement is highly evident, the younger rescued mice performed less efficiently in functional tests than normal animals (Fig. 2c) which could explain why most of the mortality recorded in the transgenic rescued mice occurred before P40. In fact, female mice tend to concentrate on pups that have the highest chance of survival; therefore, the mortality in the first weeks is probably due to competition, in the same litter, between the normal pups and the rescued less-fit animals.

We previously showed that ExSpeU1-binding to intronic sequences rescued different types of exon skipping defects^39^,^40^,^41^. Here we established that 70K and stem loop IV represent the key structural determinants involved in the splicing rescue in the modified U1-like particle. Its 5′ tail, binding downstream of the donor site, assures that two important 70K and SL4 structural elements are loaded on pre mRNA, in non-conserved intronic sequences, only in the proximity of target exons. In contrast to previously reported modified U1-based approaches, aimed at reinforcing the binding of the U1 particle at suboptimal non-conserved 5′ splice sites^51^,^52^,^53^,^54^, binding at non-conserved intronic sequences of the particles, reduces potentially deleterious off-target alternative splicing events. Indeed, in the spinal cord, a target tissue for therapy, we found that only 12 genes changed their expression (Fig. 3a) and that, in our experimental conditions, RNA-Seq identified just one alternative splicing off-target (Fig. 3b). Cyclic nucleotide binding domain containing 2 (Cnbd2) is the only gene that show changes in both expression and splicing and codes for a putative cytoplasmic protein involved in cAMP binding and spermatogenesis. As its function is inferred only from automatic GO electronic annotation, the absence of any experimental and computation evidence do not allow to clarify its potential role in the disease. Furthermore, we did not observe changes in a group of alternative splicing events previously implicated in the SMA pathogenesis (Supplementary Fig. 4).

The U1 70K protein is indispensable for splicing rescue of severe mutations for which the exon is otherwise virtually absent in the final transcript. In fact, ExSpeU1 loop I mutants that lack the 70K- binding site have no effect on splicing (Fig. 6a,b) and silencing of 70K inhibits the splicing rescue mediated by native ExSpeU1s (Fig. 6c,d). The 70K is loaded in the proximity of the 5′ splice sites and accordingly it can promote a network of interaction on the exon and facilitate its recognition. This role in splicing has been previously described *in vitro* where 70K promotes exon definition through the interaction between its RS domain and SR-splicing factors bound to exonic splicing enhancers^9^,^11^,^12^,^55^. The 70K is not obligatory for less-severe mutations for which additional factor(s) can partially compensate for its absence (Fig. 7a–c). Interestingly, U1C remains associated with loop II mutant snRNPs (Fig. 5e,f) and this factor was previously reported to have a specific effect on weakly defined alternative spliced exons^13^,^56^. Thus, in exons with less-severe mutations that maintain a residual level of exon definition, the lack of 70K is probably compensated for, in part, by U1C. ExSpeU1s, besides inducing exon definition, can also promote definition of the intron providing, in the proximity of defective exons, the stem loop IV structure, which has been reported to bind to the U2 snRNP-specific SF3A1 protein^14^. Even if the SL4 mutant that we used here, in common with other changes introduced in this structure^48^, is processed in the nucleus to a reduced RNA size (Fig. 5b), the resulting RNA maintains the Sm-binding site (Supplementary Fig. 7) and accordingly the snRNP contains the three U1-specific proteins (U1A, 70K and U1C) along with the Y12-reactive Sm protein (Fig. 5d). These data indicate that the SL4 mutant retains the Sm binding site even if truncated, and the resulting snRNP is correctly assembled and transported to the nucleus. Interestingly, we observed that U1A is largely dispensable for all types of splicing mutations (Figs 6 and 7). These results are in agreement with earliest *in vitro* splicing data using complementary U1 (refs 57, 58, 59) but pose a question regarding the role of this protein in pre mRNA processing. Indeed, U1A-associated snRNPs may have additional non splicing-related functions. For example, when U1 snRNP binds in the intron, U1A could be specifically involved in preventing the use of cryptic polyadenylation sites^16^,^17^,^60^.

Remarkably, the ExSpeU1-mediated splicing improvement does not require endogenous U1 snRNP, as assessed by U1 decoy experiments (Supplementary Fig. 6) indicating that the U1-like particles do not act by facilitating recruitment of the endogenous U1 on the upstream 5′ splice sites. The most plausible explanation is that all the different types of mutations affecting the polypyrimidine tract, exonic regulatory elements or the 5′ splice sites reduce U1 snRNP recruitment at the donor site. ExSpeU1s targeted to downstream intronic sequences compensate the mutations in the proximity of the defective exons. This provides a functional U1-like particle that promotes exon and intron definition, mainly through 70K and stem-loop IV, respectively. In addition, functional inhibition experiments with U1 decoy suggest that the exact positioning of a U1 particle with respect to the 5′ splice sites is less restricted than previously thought. In particular contexts, it is possible that binding of normal U1 snRNP to cryptic or consensus 5′ splice sites in the intronic sequences downstream of the natural 5′ splice sites might contribute to exon definition. Bifunctional U7 constructs that carry antisense and ESE sequences were previously shown to correct SMA when introduced by germline transgenesis^28^,^29^,^30^,^31^. However, to our knowledge, the resulting U7 artificial snRNPs have not been evaluated for other gene systems or for diverse types of exon skipping defects. Indeed, U7 has been extensively used as antisense molecule to induce therapeutic exon skipping *in vivo*^61^,^62^. The ExSpeU1 strategy of loading a U1-like particle in the proximity of the 5′ splice sites, represents a more general approach for recovering exon definition. In line with this, we identified ExSpeU1s that are active on additional defective exons associated with cystic fibrosis, familial dysautonomia and epidermolysis bullosa (data not shown). In addition, the significant improvement of the SMA phenotype with a single SM25 copy suggests, as we have previously reported in cellular models, that ExSpeU1 has an efficient splicing rescue activity *in vivo*.

In conclusion our results provide for the first time the evidence that precise engineering of the U1 core spliceosomal RNA particle to make it bind to intronic sequences downstream of the donor site results in molecules that have a therapeutic potential in pathologies caused by exon-skipping mutations. Exon skipping is the most frequent type of defect caused by splicing mutations and it is the leading disease-causing defect in SMA or familial dysautonomia. Furthermore in other diseases a significant number of patients show this type of aberrant splicing. For therapeutic intervention, the U1-like particles need to be delivered to the target tissues. We have previously shown that AAV-mediated administration of ExSpeU1 in SMN2 transgenic mice recovers exon 7 inclusion in several target tissues including the brain, heart and skeletal muscle^40^. Additional approaches include delivery with lentiviral vectors for a cell-based therapy *ex vivo*^41^, or synthetic modified U1 snRNAs^63^. Through the development of the most appropriate delivery system it will be possible to translate the ExSpeU1 strategy into a reliable therapeutic opportunity.

## Methods

### Animal model

All the animals were kept in a controlled environment at 25 °C with a 12 h light/dark photoperiod. The animal care and treatment were conducted in conformity with ICGEB institutional guidelines in compliance with national and international laws and policies (EEC Council Directive 86/609, OJL 358, 12 December 1987), upon approval by the Italian Ministry of Health. The fragment used for the microinjection was prepared by cloning of SM25 ExSpeU1 cassette sequence into pcDNA5 FRT/TO expression vector (Life Technologies). The amplified, digested and purified fragment was pronuclear microinjected at Plaisant Tecnopolo Castel Romano (Rome, Italy). The transgene integration site was determined using inverse PCR as described^42^. Briefly, 1 μg of genomic DNA of ExSpeU1+/+ and ExSpeU1−/− animals was digested with *NdeI* restriction enzyme for 16 h followed by heat inactivation. Digested DNA was self-ligated using 400 U μl^−1^ T4 DNA ligase (NEB, MA, USA), followed by phenol/chloroform/isoamyl extraction and precipitation. Circular DNA was PCR-amplified using ExSpeU1 transgene specific oligos (MT65 and U1160rev) and the specific bands present in the ExSpeU1 transgenic animals were extracted and TA cloned into pGEM-T Easy Vector (Promega, WI, USA). The insert was sequenced from both directions using pUC/M13 primers and the site of insertion of the transgene was determined using BLAST algorithm against mouse genome. Obtained ExSpeU1 founders were crossed with FVB.Cg-Tg(SMN2)89Ahmb^*Smn1tm1Msd*^/J *Smn1*^+/−^ (SMA colony) mice, to obtain FVB.Cg-Tg(SMN2)89Ahmb^*Smn1tm1Msd*^/J,FVB.Cg-Tg(ExSpeU1) distinct progeny. Further crossing both SMA colony and siblings procures a sufficient number of mice to analyse the molecular genetics and phenotypic tests.

### Genotyping

Tails biopsies were incubated at 55 °C overnight in lysis buffer (0.1 M Tris pH 8.0 0.2 M NaCl, 5 mM EDTA, 0.4% SDS, proteinase K 0.2 mg ml^−1^) and DNA was precipitated with isopropanol. The ExSpeU1 transgene was amplified to determine the presence and zygosity using MT97, MT98 and MT63 primers, located in the ExSpeU1 cassette and genomic integration site. To maintain heterozygosity of the mutated Smn1 allele in an SMA colony that was used for breeding with derived ExSpeU1 transgenic animals as well as identification of SMA-affected and SMA-rescued mice, we performed screening of *Smn1* and *SMN2* alleles. The primers are listed in Supplementary Tables 1 and 35 cycles of amplification were performed as follows: 94 °C 30 s, 58 °C 30 s, 72 °C 30 s.

### Gene and protein expression analysis in animal tissues

Expression of the ExSpeU1 was determined using RT–PCR and northern blotting on RNA from organs extracted from control and SMA-rescued mice. For the RT–PCR, ExSpeU1 forward and U1 cassette reverse primers were used on DNase-treated RNA samples. Northern blotting was performed as described below. For the SMN2 exon 7 splicing patterns, we used primers listed in Supplementary Table 1. SMN proteins levels were determined by western blotting in the brain, skeletal muscle and heart of P2–3 animals using an SMN-specific antibody (ab610646,BD Biosciences, USA, 1:2,000) and the levels were normalized to GAPDH (ab8245, Abcam, USA, 1:5,000). Uncropped blots are shown in Supplementary Fig. 11

### Motor function of SMA-rescued animals

Righting reflex was monitored from P8 to P19 and was defined as a time taken to turn over and place all the four paws on a warmed surface. Maximum allowed time was set to 60 s. The four-limb hanging test was performed between P14 and P50 and the time recorded is the ability of mice to hold themselves on to the bars after the metal grid was inverted, with 60 s being the maximum time. The Rotarod test was performed between P15 and P50 using homemade equipment, which accelerates from 4 to 40 r.p.m. in 5 min. Each trial ended when the mouse fell off the rod, and latency was recorded, with a maximum time of 300 s. In all the tests, the mice were tested three times per session and the results were averaged.

### Immunohistochemistry

We analysed the number of motor neurons in the grey matter of the ventral horn of the spinal cord (Rexed lamina 9) with the neuron-specific antibody NeuN and counted neurons with a cell body larger than 30 micrometre. For each genotype, we considered —three to six mice. For each spinal cord, we counted —four to six slices that were 100 micrometres apart, to avoid double counting of the same neurons.

### RNA sequencing data generation

The spinal cord RNA of wild-type (Smn1+/+, SMN2+/+, ExSpeU1−/−) and SM25 ExSpeU1 transgenic (Smn1+/+, SMN2+/+, ExSpeU1+/−) animals was purified with the TRIreagent (Invitrogen) for mRNA sequencing on HiSeq 2500 (Illumina Inc., San Diego, CA, USA). The quality of total RNA was assessed using Agilent RNA 6000 Nano Bioanalyzer microfluidic chips and a Nanodrop UV spectrophotometer. The template DNA molecules suitable for cluster generation were prepared from 2 μg of total RNA samples using the TruSeq RNA Sample Preparation Kit v2 (Illumina Inc) according to the manufacturer's instructions. The size distribution of the libraries was estimated by electrophoresis on Agilent High Sensitivity Bioanalyzer microfluidic chips. Libraries were quantified using the KAPA Library Quantification Kit (KK4824, Kapa Biosystems, Boston, MA, USA). The libraries were pooled at equimolar concentrations and diluted before loading onto the flow cell of the HiSeq 2500 (Illumina Inc.) for both clustering and sequencing. The libraries were extended and bridge-amplified to create single sequence clusters using the TruSeq Rapid PE Cluster Kit—HS (Illumina Inc.). Amplified clusters in the flow cell were then sequenced with 100-bp paired-end reads using the TruSeq Rapid SBS Kit—HS (Illumina Inc.). Real-time image analysis and base calling were performed on the the HiSeq 2500 instrument using the HiSeq Sequencing Control Software. CASAVA software version 1.8 was used for de-multiplexing and production of FASTQ sequence files. FASTQ raw sequence files were subsequently quality checked using FASTQC software version 0.11.3 http://www.bioinformatics.bbsrc.ac.uk/projects/fastqc.

### RNAseq data analysis

Paired-end RNAseq reads were mapped to the Mus Musculus reference genome to estimate gene and exon expression levels (University of California at Santa Cruz, UCSC, mm10) by using the ultrafast universal RNA-seq aligner STAR^64^. Mapped reads for all transcript variants of a gene were combined into a single value to perform differential gene expression analysis. We used the Bioconductor packages GenomicFeatures version 1.18.7 (ref. 65) in the framework of R software version 3.1.0 to download transcript annotations available at the UCSC Genome Browser and extract rounded gene or exon counts from the STAR Mapped reads. Rounded Gene counts were used as input to perform differential gene expression analysis using Bioconductor package DESeq2 version 1.4.5 (ref. 66) to estimate the per-gene negative binomial distribution dispersion parameter. Rounded Exon counts were used as input to perform differential Exon expression analysis using Bioconductor packages DEXSeq version 1.12.2 (ref. 67). To detect outlier data after normalization, we used the R packages arrayQualityMetrix^68^ and before testing differential gene expression we dropped all genes with normalized mean counts below 10 to improve testing power while maintaining type I error rates. The estimated *P* values for each gene or exon were adjusted using the Benjamini–Hochberg method. Features with adjusted *P*<0.05 and absolute logarithmic base 2 fold change >1 were considered as having a significant altered expression as previously reported^38^.

### Ingenuity pathway analysis

RNA-SEQ data were analysed by IPA (Ingenuity System, www.ingenuity.com. Release date 2015-09-14). Differentially expressed genes were incorporated in canonical pathways and bio-functions and were used to generate biological networks. Uploaded data sets for analysis were filtered using the following cutoff definitions: one log fold change, adjusted *P* value <0.05. Core analysis was performed using the following settings: Direct and Indirect, Does not Include Endogenous Chemicals, Filter Summary: consider only relationships where confidence=High (predicted) OR Experimentally Observed. The network score was based on the hypergeometric distribution and was calculated with the right-tailed Fisher's exact test. Downstream effect analysis *P* value of overlap was calculated by the Fisher's exact test.

### Minigene constructs and splicing assay

pTBFIX exon 5 and pTBCFTR exon 13 minigenes were previously described^38^. pCI-SMN2 exon 7 was obtained from Dr Adrian Krainer (CSHL, NY, USA). Exon-specific U1 snRNAs were created by replacing the sequence between the BclI and BglII sites with oligonucleotides^39^. The mutations in the ExSpeU1 sequence were introduced by overlapping PCR. The sequences of oligonucleotides are provided in Supplementary Table 1. All the constructs were verified by the sequencing. HeLa and HEK293 cell lines were grown in Dulbecco's modified Eagle's medium with Glutamax I (Gibco) (DMEM with glutamine, sodium pyruvate, pyridoxine and 4.5 g l^−1^ glucose) supplemented with 10% fetal calf serum (Euro Clone) and Antibiotic Antimycotic (Sigma). HeLa cells grown on six-well plates were transfected with Effectene reagents (Qiagen) according to the manufacturer's protocol. HEK293 cells for nuclear extract preparation were grown in p150 and transfected with calcium phosphate. Total RNA extraction was performed after 24 h of incubation using TRIreagent (Invitrogen) and reverse transcription was performed with random primers and Moloney murine leukemia virus enzyme (Invitrogen)^38^. The Alpha2,3 and Bra2 oligonucleotides were used for amplification of pTB-based minigenes: PCIfor and E8–75rev oligonucleotides for pCI-SMN2. PCR products were resolved by 2% agarose gel electrophoresis. The quantification of exon inclusion was performed using the ImageJ software.

### siRNA and U1 snRNA decoy

Silencing of U1A, U1C and 70K were performed by transfecting siGenome Smart pool (Dharmacon) RNAi oligos (SNRPC, SNRNR70, SNRPA sequences in Supplementary Table 1) using Oligofectamine Reagent (Invitrogen) in Hela cells according to the manufacturer's instructions. After 48 h of siRNA treatment, the cells were transfected with minigenes and corresponding ExSpeU1s. Subsequently, the cells were divided and a fraction was used for protein analysis for determination of silencing efficiency by western blot, using monoclonal antibodies against U1C (4H12, ab122901 Abcam, 1:1,000), U1-70K (ab83306, Abcam. 1:1,000) and U1A (ab55751, Abcam, 1:5,000). Uncropped blots are shown in Supplementary Fig. 11. A fraction was used to extract total RNA for transcript analysis. U1 snRNA 5′ blocking was achieved by co-transfection of the D1 plasmid (courtesy of X. Roca) as described in Supplementary Fig. 2 and according to^44^. D3 plasmid carrying sequence which was previously reported not to bind to U1 snRNA was used as control^44^.

### Northern blot analysis of snRNA

Northern analysis of U1 snRNA was carried out as follows. Total RNA was prepared and separated on 8% polyacrylamide-7 M urea gels, then transferred to GeneScreen Plus Hybridization Transfer Membranes (PerkinElmer) using a semi-dry transfer apparatus. Northern hybridization was performed using the ^32^P 5′-end-labelled oligonucleotide probes listed in Supplementary Table 1. U6 snRNA was used as loading control. The quantification was performed using OptiQuantTM software with a Cyclone Phosphor Imager (PerkinElmer).

### EMSA and RNase H protection assay

EMSA was performed using RNA oligos synthesized by Sigma, Life Science and radio-labelled using [γ-^32^P] ATP and T4 polynucleotide kinase (New England Biolabs). Binding reactions containing labelled RNA probes, together with nuclear extracted obtained from transfected HEK-293 cells, were performed in 1 × binding buffer (10 mM NaCl_2_, 10 mM Tris pH 8.0, 2 mM MgCl_2_, 5% glycerol and 1 mM DTT) for 60 min at room temperature before electrophoresis on a 8% polyacrylamide native gel at 100 V for 1.5 h in 0.5 × Tris borate/EDTA buffer at 4 °C. Super-shifts were obtained by adding antibodies specific to U1A, U170K and U1C. A pre-run of the gel (approximately 20 min) was performed before the samples were loaded. Following electrophoresis, the gels were dried on 3MM Whatman paper and exposed on a Cyclone Phosphor screen (Packard). For RNase H protection assays, whole-cell extracts in RSB-100 buffer were incubated with 5 μM antisense DNA oligonucleotide complementary to U1snRNA (5′-CAGGTAAGTAT-3′) and 1.5 U RNase H (Promega) for 30 min at 30 °C. Total RNA was extracted and analysed by northern blotting on a 12% polyacrylamide gel using an internal U1 probe (5′-CAAATTATGCAGTCGAGTTTCCCACATTTG-3′).

### Affinity purification of U1 snRNP

The affinity purification of U1 snRNPs from HEK-293 nuclear extracts was performed according to ref. 69. The 3′-biotinylated 2′-*O*-methyl antisense RNA oligonucleotides directed against the 5′ end of the U1 snRNA were incubated with 30 μl HeLa nuclear extract in a total volume of 100 μl binding buffer (20 mM HEPES/KOH pH 7.5, 100 mM KCl, 10 mM MgCl_2_, 0.01% NP-40, 1 mM DTT) for 1.5 h at room temperature. Bound material was pulled down via NeutrAvidin agarose beads (Thermo Scientific) for 2 h at 4 °C and washed several times in (20 mM HEPES/KOH pH 7.5, 200 mM KCl, 10 mM MgCl_2_, 0.01% NP-40, 1 mM DTT). Affinity-selected U1 snRNA complexes were examined by western blot using U1 specific antibodies.

### RNA immunoprecipitation

Protein A/G PLUS-Agarose beads (40 μl; Life Technologies) were incubated with antibodies against U1A, U1C and U1-70K for 2 h at 4 °C. The excess of antibody was washed with buffer (50 mM Tris pH 7.4, 150 mM NaCl, 0,05% ND40). Nuclear extract was prepared from Flp-In-293 cells transfected with ExSpeU1 or mock and was incubated with antibody-coated beads at 4 °C for 4 h. The protein complexes were eluted by boiling with Laemmli buffer and analysed by western blot. RNA was extracted by Trizol using standard procedure and analysed by northern blot probing against U1snRNA and ExSpeU1.

### 3′ adaptor ligation mediated PCR

Total RNA extracted from cells transfected with ExSpeU1 SM25 L4mut was ligated with 3′ RNA adaptor (5′-pUCGUAUGCCGUCUUCUGCUUGidT-3′). Next RT–PCR was performed using an oligo specific for ExSpeU1 SM25 (5′–ATGTTTTCATTCTAAGT-3′). PCR products were purified, cloned in pUC19 vector and 10 independent clones were sequenced.

## Additional information

**Accession codes:** RNA-Seq data have been deposited in Sequence Read Archive (SRA) under accession code Bio Project ID PRJNA305817.

**How to cite this article:** Rogalska, M. E. *et al*. Therapeutic activity of modified U1 core spliceosomal particles. *Nat. Commun.* 7:11168 doi: 10.1038/ncomms11168 (2016).

## Supplementary Material

Supplementary Information Supplementary Figures 1-11 and Supplementary Table 1
