# Supplementary material for: Therapeutic activity of modified U1 core spliceosomal particles
Source: Nat Commun. 2016 Apr 4;7:11168. doi: 10.1038/ncomms11168 (PMC4822034; doi:10.1038/ncomms11168)
Supplement: Supplementary Information — Supplementary Figures 1-11 and Supplementary Table 1 [file ncomms11168-s1.pdf]

a

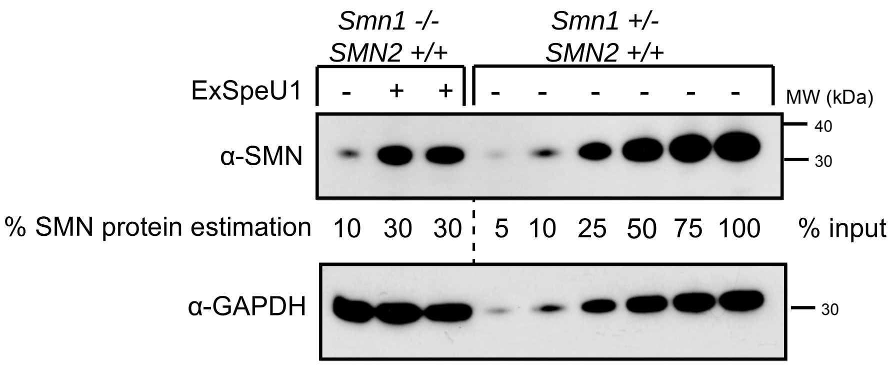

b

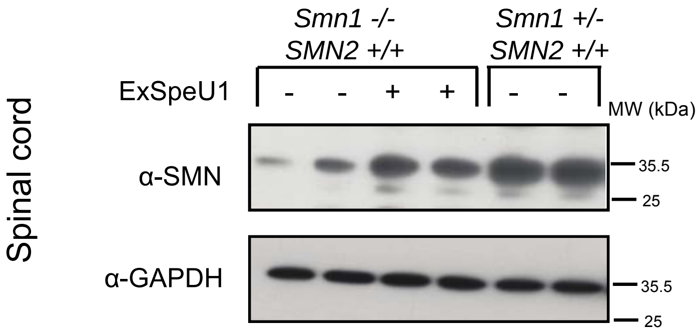

Supplementary Figure 1. SMN protein expression levels in brain and spinal cord.

a SMN protein levels estimation by Western Blotting in brain **b** SMN protein levels detected by Western Blotting in Spinal cord. Analysis is performed on SMA-affected (Smn1-/-, SMN2+/+), SMA-rescued (Smn1-/-, SMN2 +/+; ExSpeU1 +/-), and control animals (Smn1+/-, SMN2+/+, ExSpeU1+/-) at P2-3. alfa-GAPDH was used as internal control.

a

	Gene ID	log2FoldChange	pvalue
Up regulated	1700001G11Rik	-2.37	1.49E-09
	Rgp1	-2.13	7.25E-07
	9330162012Rik	-2.12	1.03E-06
	Cnbd2	-1.99	9.50E-06
	Nt5c2	-1.98	4.80E-06
	Mrpl48	-1.86	5.61E-05
	Ptges3l	-1.73	1.12E-04
	Armc6	-1.73	7.02E-05
	Grin3b	-1.69	7.89E-06
	Elf1	-1.45	6.94E-05
	Gabpa	-1.33	3.49E-05
Down	Ubc	1.61	1.78E-04

b

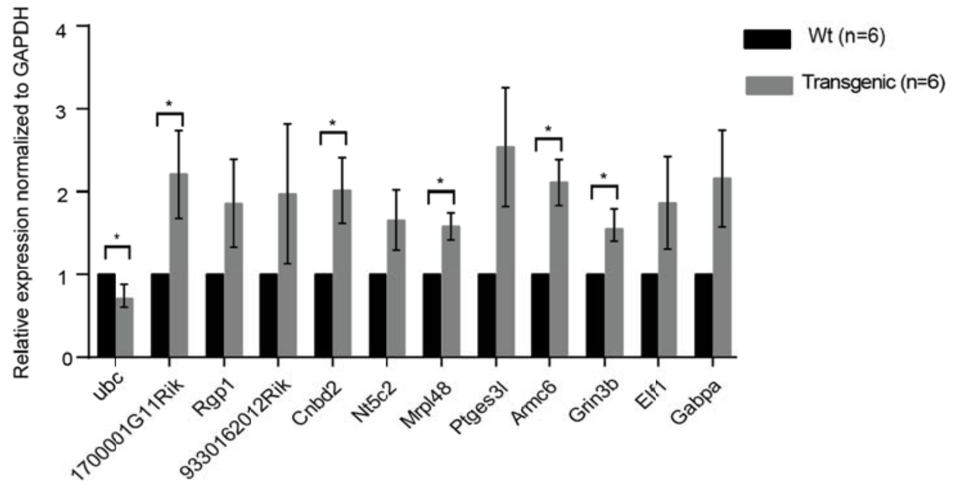

c

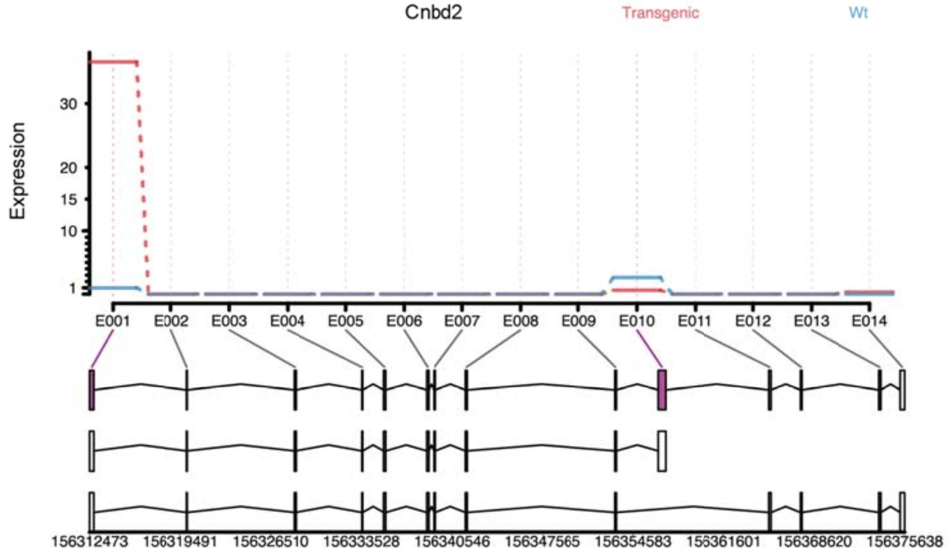

Supplementary Fig. 2 Candidate genes and exons identified in RNAseq

a Transcriptional changes in spinal cord of wild type (Smn1+/+, SMN2+/+, ExSpeU1-/-) and SM25 ExSpeU1 transgenic (Smn1+/+, SMN2+/+, ExSpeU1+/-) animals analyzed by applying a threshold of Log2FC>±2 and a multiple testing correction (MTC) *P* value <0.05. Using this threshold we identified 11 genes that were upregulated and 1 that was downregulated. **b** Validation of transcriptional changes using qRT-PCR. Graph represents relative expression fold changes in transgenic (Smn1+/+, SMN2+/+, ExSpeU1+/-) and wild type (Smn1+/+, SMN2+/+, ExSpeU1-/-) animals. Wt mice were set to 1. Data represent the mean ± SD in 6 animal per group (Student *t*-test * p ≤ 0.05) **c** *Cnbd2* exon plot. Changes shown are represented as log2-fold increases in exon reads relative to wild type and SM25 ExSpeU1 transgenic animals.

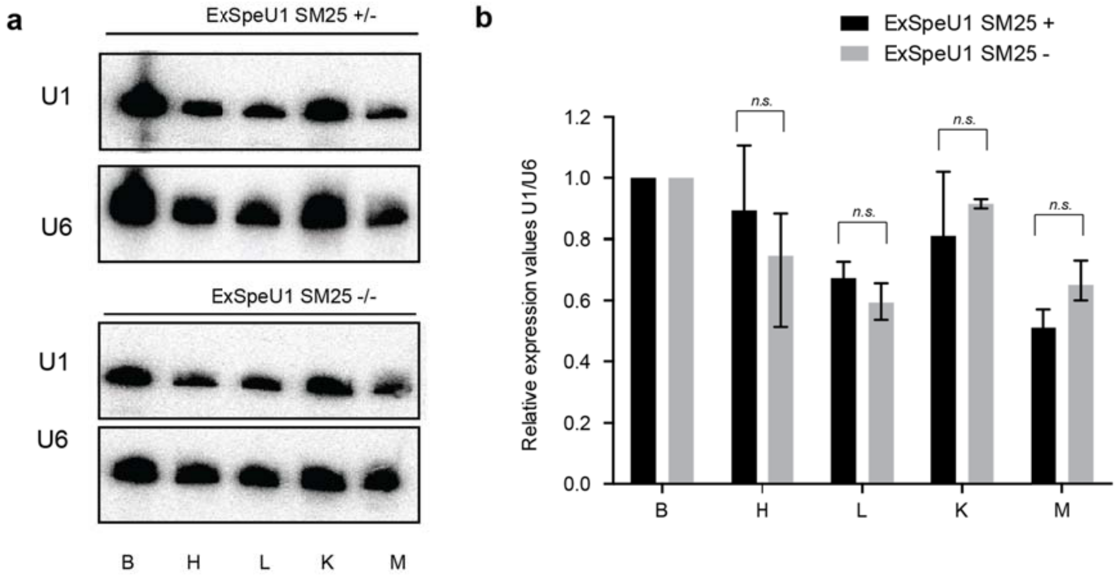

Supplementary Fig. 3. ExSpeU1 SM25 expression does not affect endogenous U1/U6 snRNA levels in transgenic mice.

a Northern blot hybridization of RNA extracted from five mice tissues of transgenic (Smn1+/+, SMN2+/+, ExSpeU1+/-) and wild type mice (Smn1+/+, SMN2+/+, ExSpeU1-/-) probed for U1 and U6. The histogram shows the quantification of U1/U6 ratio estimated by OptiQuantTM software. Brain ratio was set to 1. Data represent the mean ± SD of expression in four animals (Student t-test, n.s. not significant).

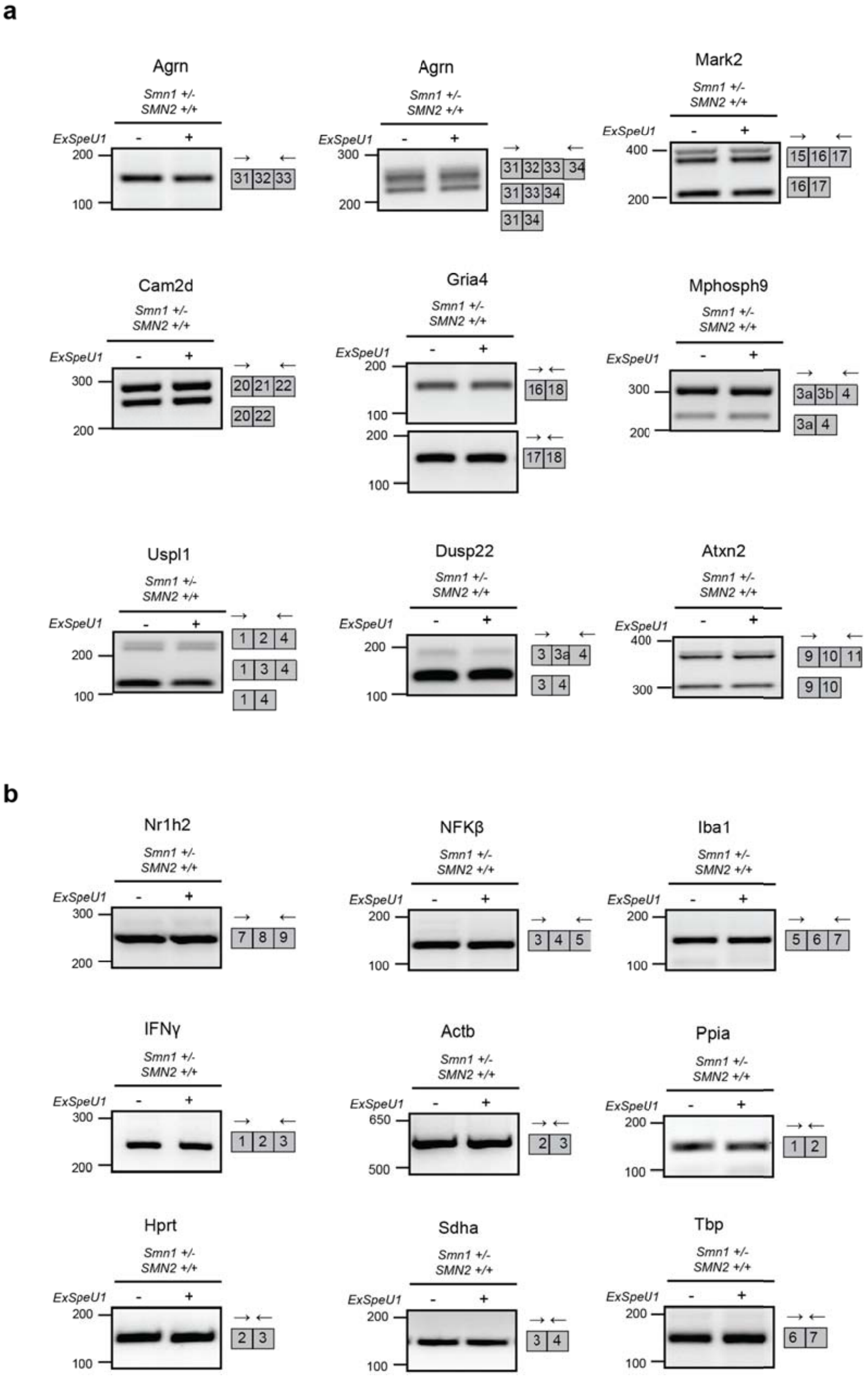
a
Agrn
Smn1 +/-
SMN2 +/+
ExSpeU1
-
+
200
100
31 32 33
Agrn
Smn1 +/-
SMN2 +/+
ExSpeU1
300
200
31 32 33 34
31 33 34
31 34
Mark2
Smn1 +/-
SMN2 +/+
ExSpeU1
400
200
15 16 17
16 17
Cam2d
Smn1 +/-
SMN2 +/+
ExSpeU1
300
200
20 21 22
20 22
Gria4
Smn1 +/-
SMN2 +/+
ExSpeU1
200
100
16 18
200
100
17 18
Mphosph9
Smn1 +/-
SMN2 +/+
ExSpeU1
300
200
3a 3b 4
3a 4
Uspl1
Smn1 +/-
SMN2 +/+
ExSpeU1
200
100
1 2 4
1 3 4
1 4
Dusp22
Smn1 +/-
SMN2 +/+
ExSpeU1
200
100
3 3a 4
3 4
Atxn2
Smn1 +/-
SMN2 +/+
ExSpeU1
400
300
9 10 11
9 10
b
Nr1h2
Smn1 +/-
SMN2 +/+
ExSpeU1
300
200
7 8 9
NFKβ
Smn1 +/-
SMN2 +/+
ExSpeU1
200
100
3 4 5
Iba1
Smn1 +/-
SMN2 +/+
ExSpeU1
200
100
5 6 7
IFNγ
Smn1 +/-
SMN2 +/+
ExSpeU1
300
200
1 2 3
Actb
Smn1 +/-
SMN2 +/+
ExSpeU1
650
500
2 3
Ppia
Smn1 +/-
SMN2 +/+
ExSpeU1
200
100
1 2
Hprt
Smn1 +/-
SMN2 +/+
ExSpeU1
200
100
2 3
Sdha
Smn1 +/-
SMN2 +/+
ExSpeU1
200
100
3 4
Tbp
Smn1 +/-
SMN2 +/+
ExSpeU1
200
100
6 7

Supplementary Fig. 4 Profile of alternative and constitutive splicing events in spinal cord of Wt and transgenic mice.

a Alternative splicing events detected by RT-PCR reaction in RNA extracted from spinal cords of transgenic (Smn1+/+, SMN2+/+, ExSpeU1+/-) and wild type (Smn1+/+, SMN2+/+, ExSpeU1-/-) adult animals. Three animals for each group were analysed and no differences were detected. Pictures are representative of obtained pattern of splicing. **b** Constitutive splicing events were analized as in **a**. No differences were detected between transgenic and wild type animals.

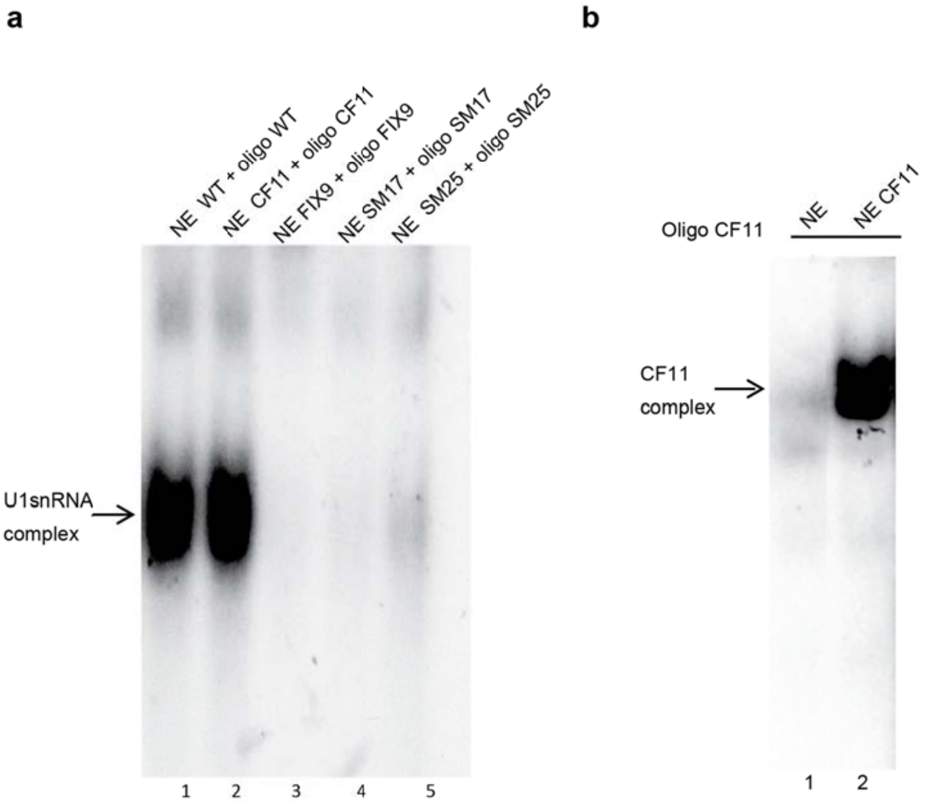

Supplementary Fig.5. ExSpeU1 snRNP analysis in EMSA.

a Complex formation occurs only for ExSpeU1 CF11. EMSA assay was performed on nuclear extract prepared from untreated HEK293 cells and cells transfected with different ExSpeU1s and incubated with corresponding labelled RNA oligo (WT, CF11, FIX9, SM17 and SM25). **b** CF11 oligonucleotide does not show any complex formation in untreated cells.

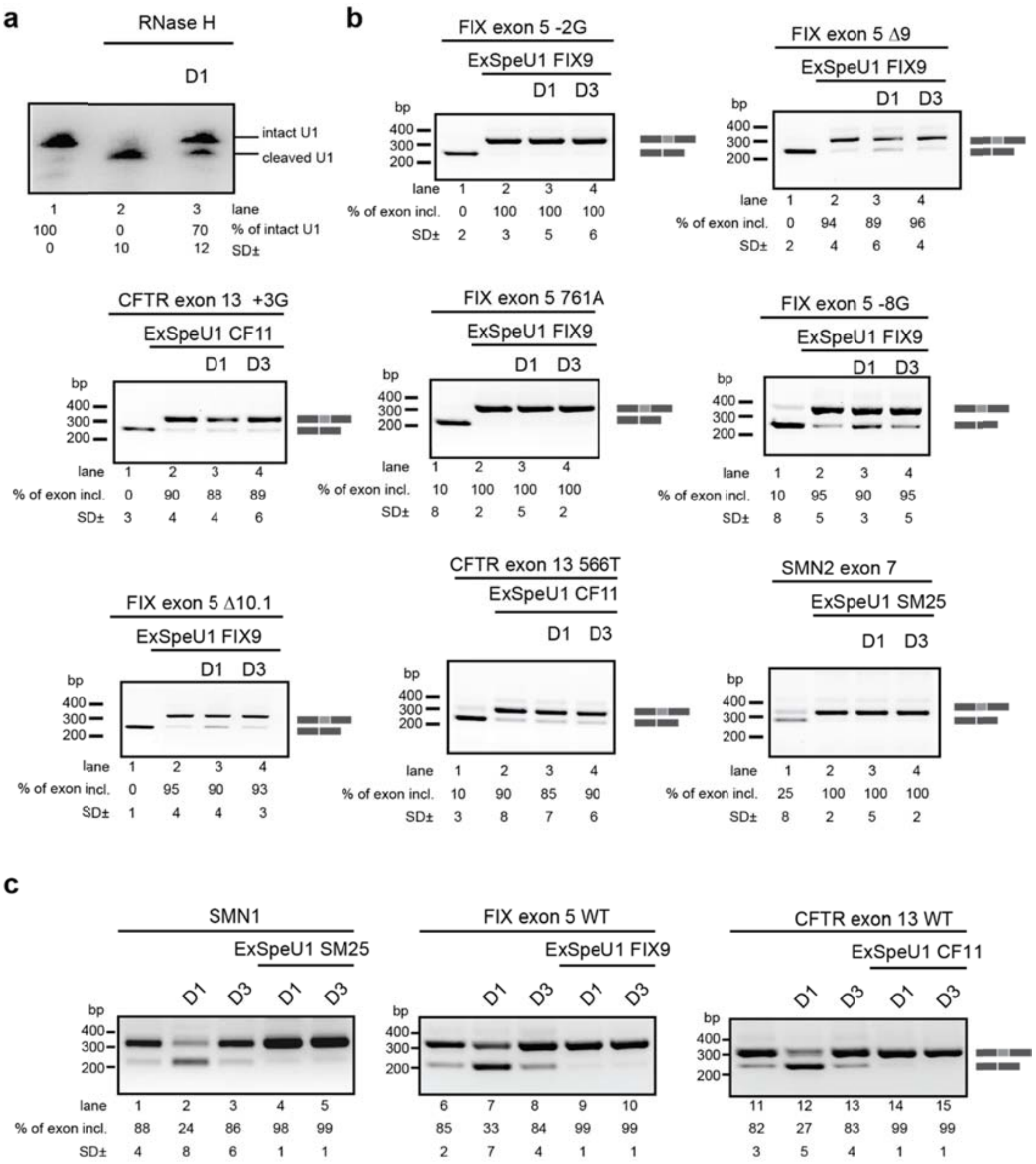

Supplementary Fig. 6. ExSpeU1-mediated splicing rescue does not require endogenous U1 snRNP.

a D1 treatment masks ~70% of endogenous U1snRNP . HeLa cells were transfected with 850ng of D1 or control D3 plasmids for 24 hrs. RNase H protection assay was performed using total cell extracts and U1 snRNA was detected by northern blotting

using internal probe. Quantification was performed by OptiQuantTM software. Data represent the mean ± SD of 3 experiments. **b** Analysis of spliced transcripts of mutated (**b**) and wild type (**c**) minigenes co-transfected with ExSpeU1 and decoy plasmid. HeLa cells were transfected with 850ng of D1 or D3 plasmids and with 500ng of corresponding ExSpeU1s as indicated. The splicing pattern was evaluated by RT-PCR. Percentage of exon inclusion was quantified by ImageJ. Data represent the means ± SD of three independent experiments done in duplicate.

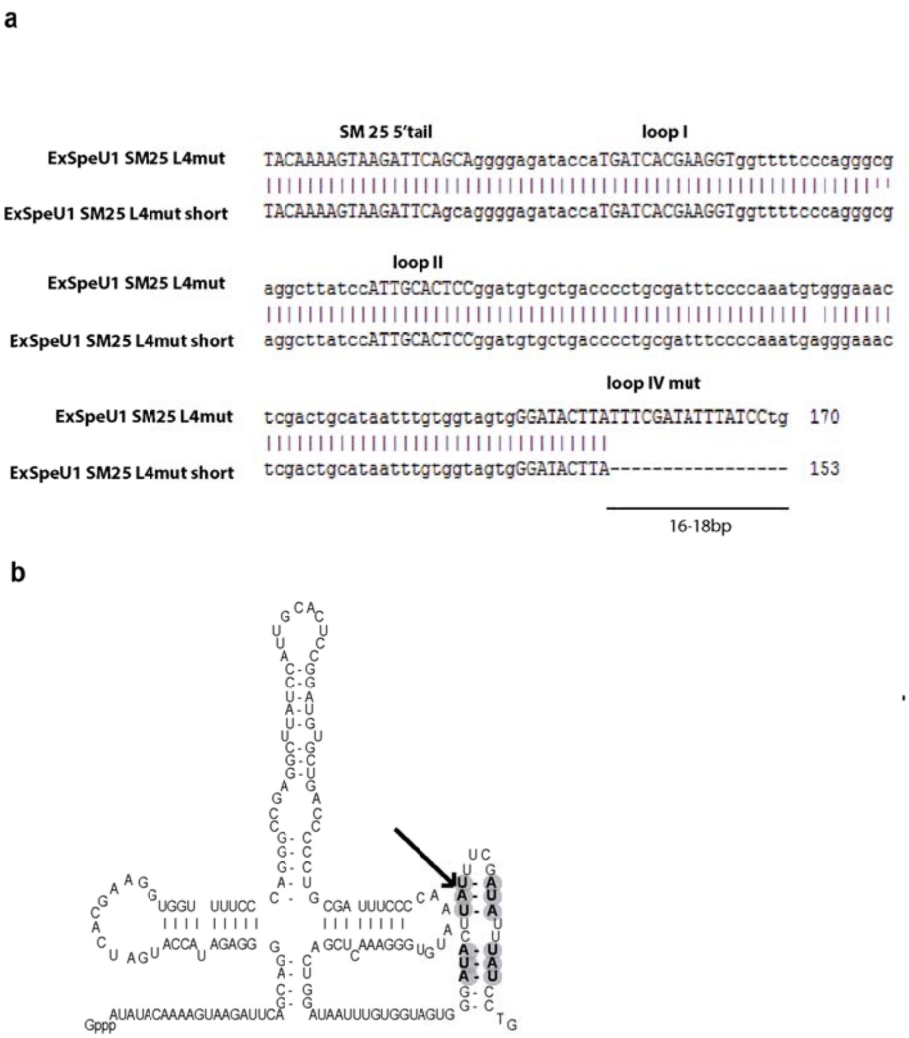

Supplementary Fig. 7. The SM25 SL4 mutant is processed to a truncated U1 RNA isoforms that lacks the last ~16-18 nucleotides.

a Sequence alignment of the two ExSpeU1 SM25 L4mut isoforms. To determine the nucleotide composition of two full length and short isoforms identified in Northern Blotting (Fig 5 b) we performed 3'adapter ligation with total RNA. RT-PCR was performed using primer specific for ExSpeU1 SM25 and the 3'adapter. PCR products were cloned into pUC vector and ten positive clones were sequenced. **b** Schematic representation of ExSpeU1 SM25 L4 mutant secondary structure. Arrow indicates the end of the shorter isoform.

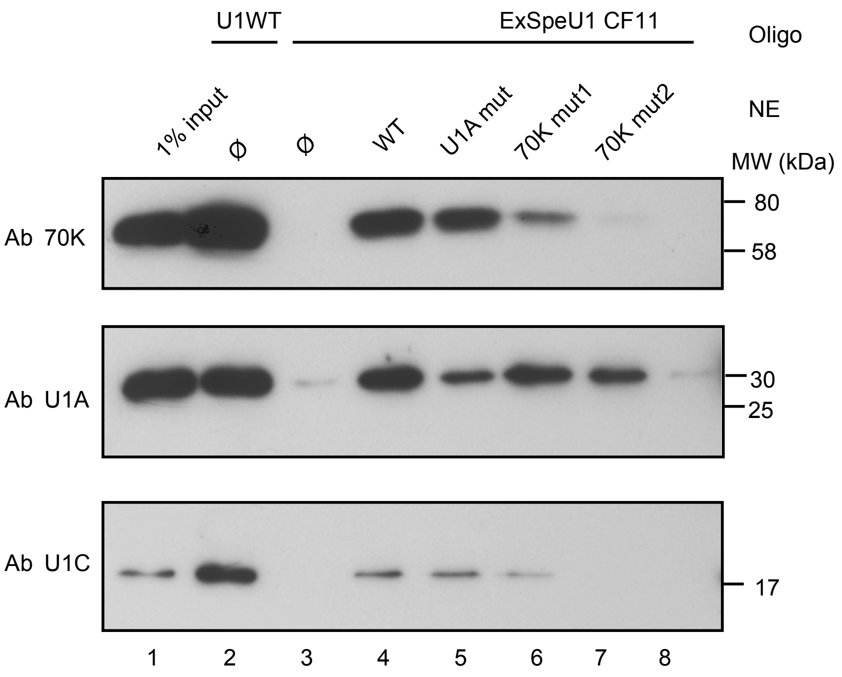

Supplementary Fig. 8. Affinity purification of ExSpeU1 CF11 mutants.

ExSpeU1 CF11 was affinity-purified from nuclear extract prepared from HEK293 cells transfected with WT, U1A, 70K, or MS2 mutants. Proteins were analyzed on 8% SDS-PAGE followed by WB to detect 70K, U1A and U1C. For uncropped gels see Supplementary Figure 16.

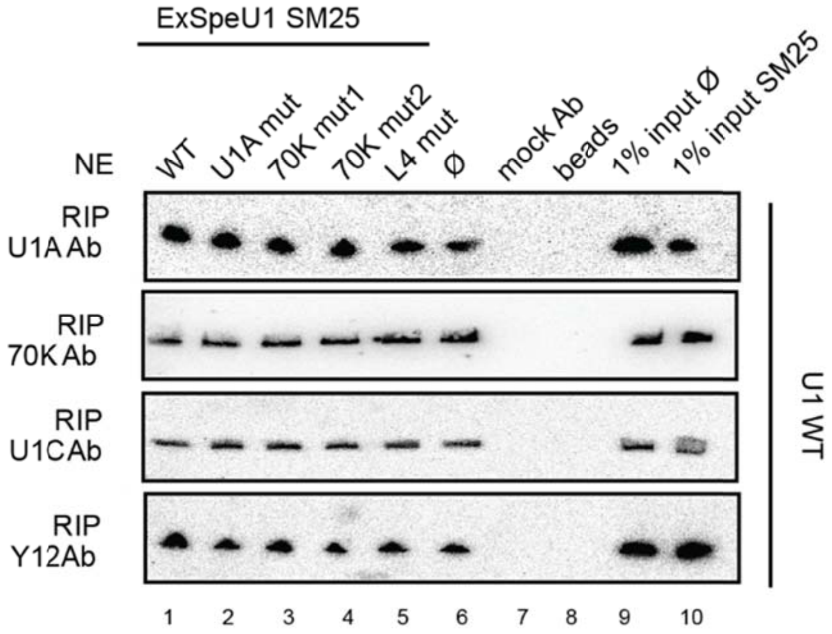

Supplementary Fig. 9. Detection of endogenous U1 snRNA in RNA-IP experiments.

Membranes from RNA immunoprecipitation assay (Fig. 5f) were stripped and re-probed for U1 snRNA, as an internal control.

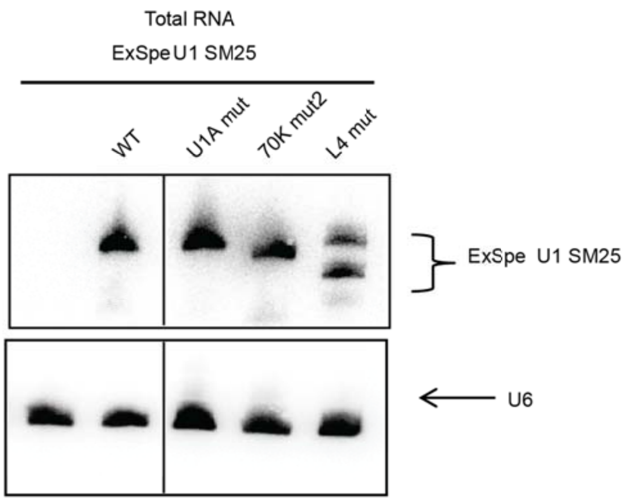

Supplementary Fig. 10. ExSpeU1 SM25 mutants are expressed in the same amount as wild-type particle in co-transfection experiments.

Northern blot hybridization of RNA extracted from cells transfected with 250 ng of ExSpeU1 SM25 and 500 ng of its mutated variants. Membranes were probed for U1 ExSpeU1 SM25 and U6 snRNA as a loading control.

Fig 1c

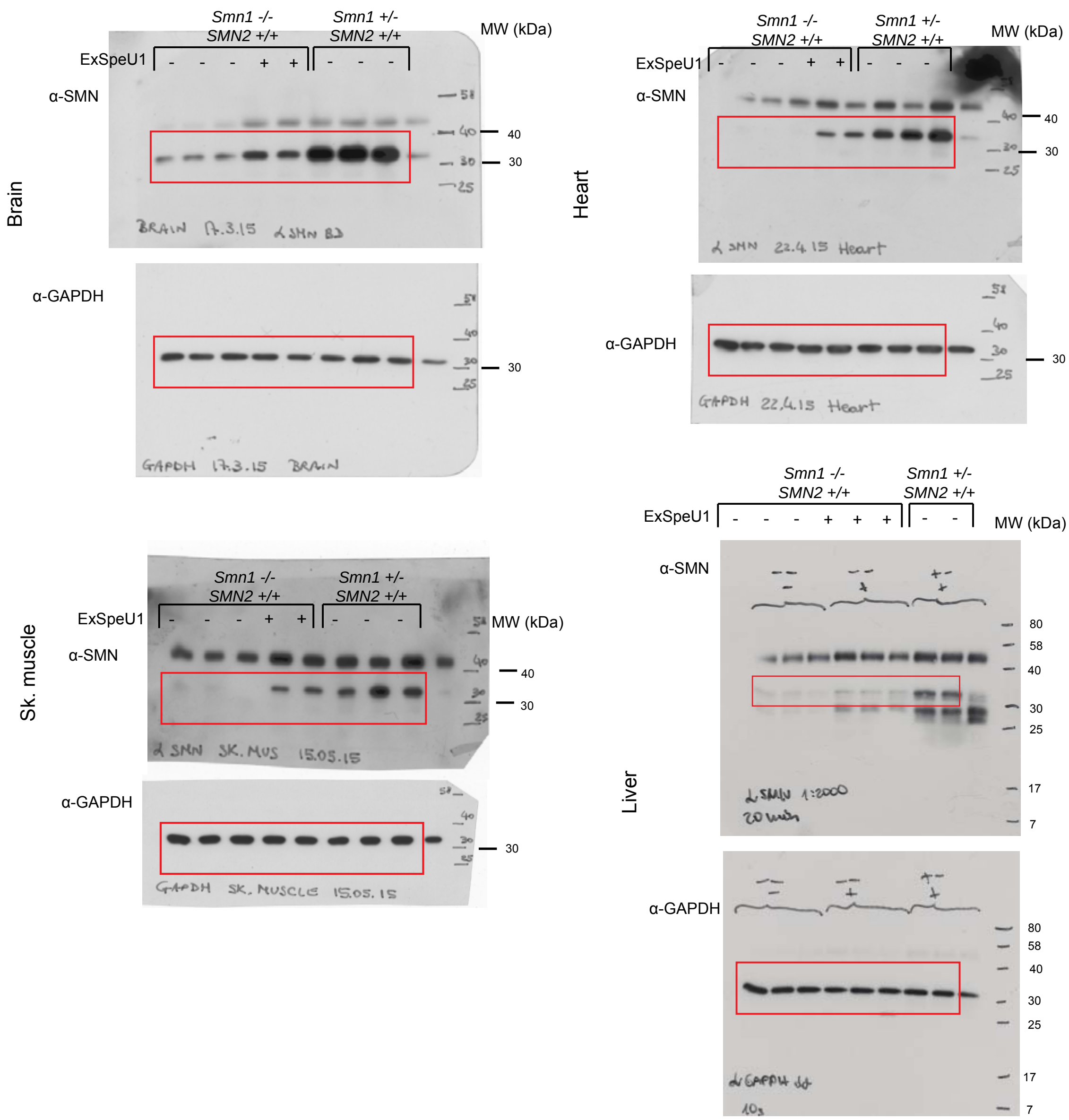

Supplementary Fig. 11a Uncropped gels related to Fig 1

Uncropped gels Fig 4

Fig 4b

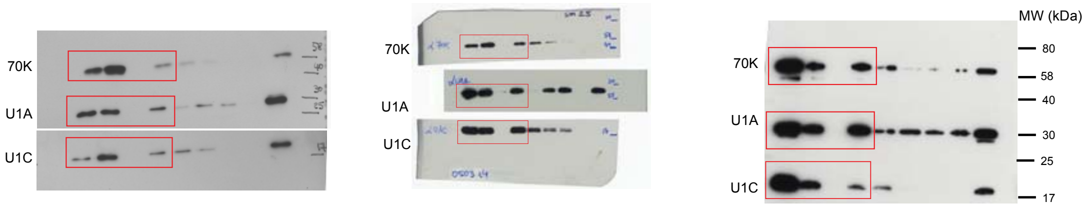

Fig 4c

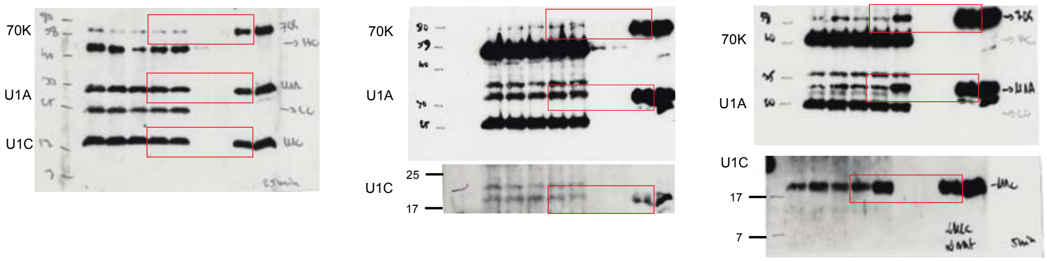

Supplementary Fig. 11b. Uncropped gels related to Fig 4

Uncropped gels Fig 5

Fig 5c

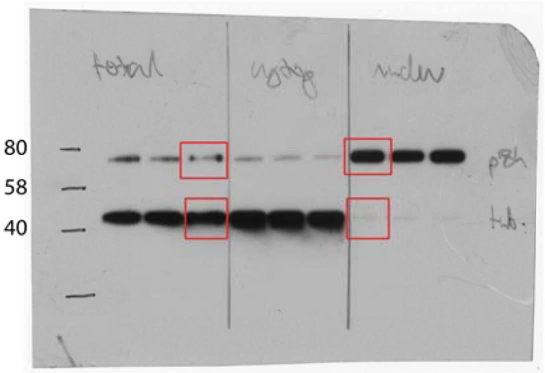

Fig 5e

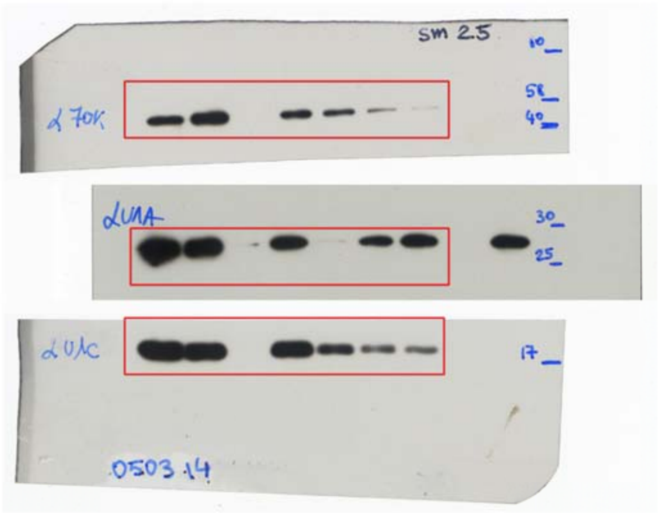

Supplementary Fig. 11c. Uncropped gels related to Fig 5

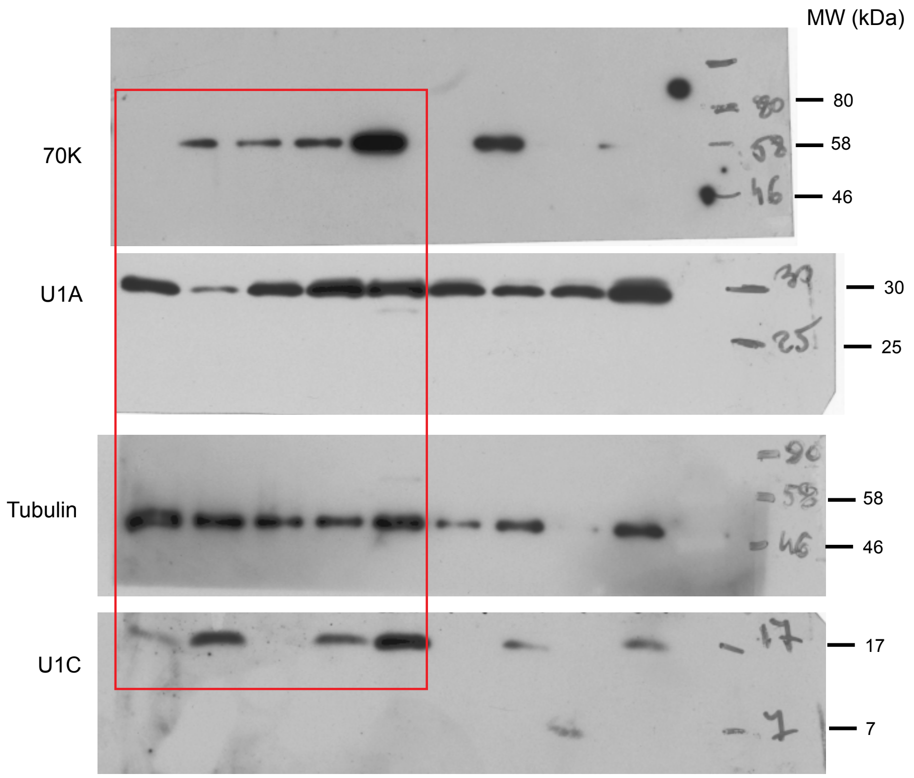

Supplementary Fig. 11d. Uncropped gels related to Fig 6

Supplementary Fig 1a

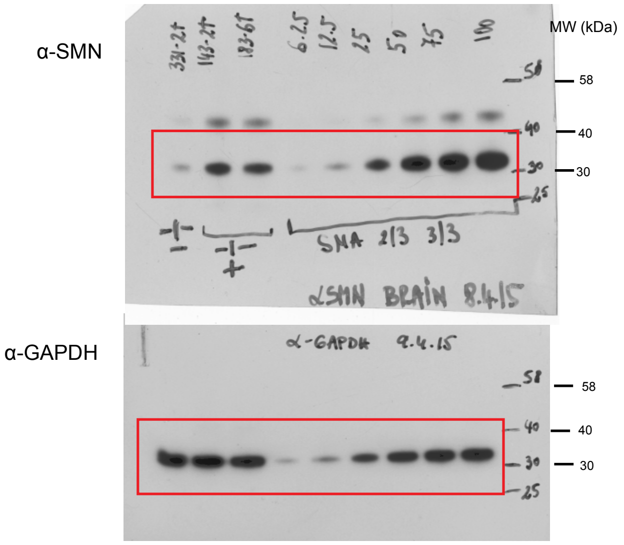

Supplementary Fig 1b

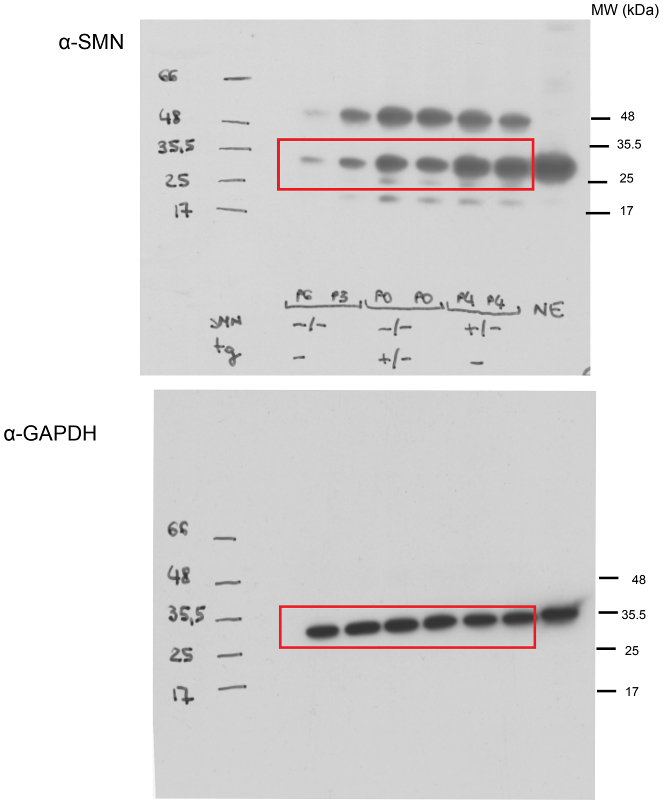

Supplementary Fig. 11e. Uncropped gels related to Supplementary Fig 1

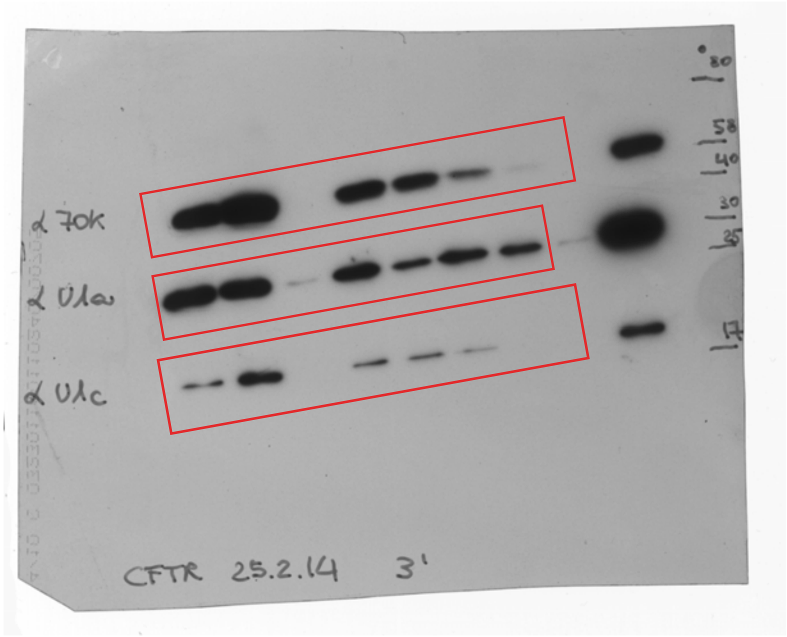

Supplementary Fig. 11f. Uncropped gels related to Supplementary Fig. 8

Supplementary Table 1: Primers and oligonucleotides

Gene to identify	PCR primer name	5'-3' sequence
**Primers used for cloning**		
U1 mutants	U1Amutfor	GAGGCTTATCCCTTAGACTCCGGATGTGC
	U1Amutrev	CTCCGAATAGGGAATCTGAGGCCTACACG
	L4for	CATAATTTGTGGTAGTGATATACTTATTTCGATATTTATA TTGACTTTCTGGAG
	L4rev	CTCCAGAAAGTCAATATAAATATCGAAATAAGTATATCA CTACCACAAATTATG
	U1215for	CGTGCTTCACCACGAACCAGTTCC
	SP6rev	ATTTAGGTGACACTATAG
ExSpeU1 CF11	CF11for	GATCTCATAAGTAAGGTATTCAGCAGGGGAGATACCAT
	CF11rev	GATCATGGTATCTCCCCTGCTGAATACCTTACTTATGA
	CF1170Km1	CCCAAGATCTCATAAGTAAGGTATTCAGCAGGGGAGAT ACCATTGACACG
	CF1170Km2	CCCAAGATCTCATAAGTAAGGTATTCAGCAGGCCTGAT ACACCATCAGGGTTCAGGCAGGGCGAGGC
ExSpeU1 FIX9	FIX9for	GATCTCATTCTTATTCAGGCAGGGGAGATACCAT
	FIX9rev	GATCATGGTATCTCCCCTGCCTGAATAAGAATGA
	FIX9for70Km1	CCCAAGATCTCATTCTTATTCAGGCAGGGGAGATACCA TTGACACG
	FIX9for70Km2	CCCAAGATCTCATTCTTATTCAGGCAGGCCTGATACAC CATCAGGGTTCAGGCAGGGCGAGGC
ExSpeU1 SM25	SM25for	GATCTCATATACAAAAGTAAGATTCAGCAGGGGAGATA CCAT
	SM25rev	GATCATGGTATCTCCCCTGCTGAATCTTACTTTTGTATA TGA
	SM2570Km1	CCCAAGATCTCATATACAAAAGTAAGATTCAGCAGGGG AGATACCATTGACACG
	SM2570Km1	CCCAAGATCTCATATACAAAAGTAAGATTCAGCAGGCC TGATACACCATCAGGGTTCAGGCAGGGCGAGGC
**Primers used for minigene splicing assay**		
pTB	Alfa2-3	CAACTTCAACTCCTAAGCCACTGC
	Bra2	GTCACCAGGAAGTTGGTTAAATCA
SMN	PCIfor	GACTCACTATAGGCTAGCCTCG
	E8-75rev	AAGTACTTACCTGTAACGCTTCACATTCCAGATCTGTC
**Animal model**		
ExSpeU1 genotyping	MT97	CCTGTAAGGATGTGTGAATG
	MT98	ATACTAGCCATCTCTCTTGG
	MT63	TACACTTGCCAGCGCCCTAG
Smn1 genotyping	oIMR7208	CTCCGGGATATTGGGATTG
	oIMR7210	GGTAACGCCAGGGTTTTCC
	oIMR7271	TTTCTTCTGGCTGTGCCTTT
SMN2 genotyping	oIMR5065	CTGACCTACCAGGGATGAGG
	oIMR5066	GGTCTGTTCTACAGCCACAGC
	oIMR5067	CCCAGGTGGTTTATAGACTCAGA
SMN2 RT-PCR	MT26	GATGCTGATGCTTTGGGAAGT
	MT28	TCTGATCGTTTCTTTAGTGGTGTC
Inverse PCR	MT65	GCCCTGTAGCGGCGCATTAA
	U1160rev	GGAAAGCGCGAACGCAGTC
**Oligo used for silencing (siGNOME Smart pool siRNA)**		
70K		GAGAGUGAAUUAUGACACA<br>GUACGGACCUAUCAAAAGA<br>GCCGUACAUUCGAGAGUUU<br>GAGACAUGCACUCCGCUUA
U1A		GCCCCUAACCACACUAUUUA

	GCCCCUAACCACACUAUUUA<br>UCAAGAAGGAUGAGCUAAA<br>UGACAAACCUAUGCGUAUC
U1C	AUAAAAGACUAUUAUCAGA<br>AAACAACGGCUGCAUUUCA<br>AAAGAUACCUCCUACUCCA<br>UAUUGUGACUACUGCGAUA
**RNA oligos complementary to 5' tails of studied U1**	
WT	3' UAUGAAUGGUCCG 5'
CF11	3' UAUUCAUUCCAUAAGU 5'
FIX9	3' UAAGAAUAAGAC 5'
SM25	3' UAUGUUUUCAUUCUAAGU 5'
**Primers used for RNAseq validation**	
GAPDH	F 5'AAGGGCTCATGACCACAGTC 3'<br>R 5'ACACATTGGGGGTAGGAACA 3'
Elf1	F 5' CCAAGTATTAGGACTATACAGG 3'<br>R 5' GGCTATAACCGTTGTGAGTG 3'
Gapda	F 5' CCGGGACCTTACCCTGCTAC 3'<br>R 5' TCTCAGTCCCGTCGATCTCA 3'
Grin3	F 5' GAGGTCTGTGCCAGGTTCTG 3'<br>R 5' ATGGTCTCCAGGGGACTAGC 3'
Ubc	F 5' GCCCAGTGTTACCACCAAGA 3'<br>R 5' CCCATCACACCCAAGAACA 3'
Rgp1	F 5' ACCAGCTTCTCTCTCCCCAT 3'<br>R 5' AATGGCGGCAGAAGTACCAA 3'
Mrpl48	F 5' GCTGGGTGTGTGGACCAATA 3'<br>R 5' TTTGGGCTCCTGCACTTTCA 3'
Cndb	F 5' GCTGGGTGACGAAGTTCAGA 3'<br>R 5' CACAGTTGCCCTGGCAGATA 3'
Ptges3l	F 5' CGTGTTCAGCTGCAGGAAT 3'<br>R 5' TCAGCTCCACCTCATCGTCTC 3'
Nt5c2	F 5' AGCGACTGCGAGCGGAAG 3'<br>R 5' CACTCCAGGAGGTCGTCATCT 3'
Armc6	F 5' AGG CCT GAA GGT GCT CAT TG 3'<br>R 5' CGA GGT CCT GGT GGT CAT TG 3'
1700001G11Rik	F 5' GCGTTGTCCCTGCTGAAGAT 3'<br>R 5' GACAGATCCTTCCGCCTTGG 3'
9330162012Rik	F 5' ATGTCGGATGCTCACCTGTC 3'<br>R 5' GAACCGATCACTGAGCCACA 3'
**Primer used for splicing analysis in mice spinal cord**	
Nr1h2	F 5' TTGTGGACTTTGCCAAGCAG 3'<br>R 5' GCGATAAGCAAGGCATACTC 3'
NFKβ	F 5' AGCAGGACATGGGATTTCAG 3'<br>R 5' TAGGTGGATGATGGCTAAGTG 3'
Iba1	F 5' AATGATGAGGATCTGCCGTC 3'<br>R 5' AGTCAGAGTAGCTGAACG 3'
IFNγ	F 5' CACGGCACAGTCATTGAAAG 3'<br>R 5' TTGCTGATGGCCTGATTGTC 3'
Actb	F 5' CATGTTTGAGACCTTCAACA 3'<br>R 5' ATCTCCTTCTGCATCCTGTC 3'
Ppia	F 5' CAGACGCCACTGTCGCTT 3'<br>R 5' TGTCTTTGGAACTTTGTCTGCAA 3'

Hprt	F 5’ TTGCTCGAGATGTCATGAAGG 3’<br>R 5’ TGAGAGATCATCTCCACCAAT 3’
Sdha	F 5’ GCTTGCGAGCTGCATTTGG 3’<br>R 5’ CATCTCCAGTTGTCCTCTTCC 3’
Tbp	F 5’ CTCAGTTACAGGTGGCAGCA 3’<br>R 5’ ACCAACAATCACCAACAGCA 3’
